# High-affinity anti-Arc nanobodies provide tools for structural and functional studies

**DOI:** 10.1371/journal.pone.0269281

**Published:** 2022-06-07

**Authors:** Sigurbjörn Markússon, Erik I. Hallin, Helene J. Bustad, Arne Raasakka, Ju Xu, Gopinath Muruganandam, Remy Loris, Aurora Martinez, Clive R. Bramham, Petri Kursula

**Affiliations:** 1 Department of Biomedicine, University of Bergen, Bergen, Norway; 2 VIB-VUB Center for Structural Biology, Vlaams Instituut voor Biotechnologie, Brussels, Belgium; 3 Department of Bioengineering Sciences, Structural Biology Brussels, Vrije Universiteit Brussel, Brussel, Belgium; 4 Faculty of Biochemistry and Molecular Medicine & Biocenter Oulu, University of Oulu, Oulu, Finland; Griffith University, AUSTRALIA

## Abstract

Activity-regulated cytoskeleton-associated protein (Arc) is a multidomain protein of retroviral origin with a vital role in the regulation of synaptic plasticity and memory formation in mammals. However, the mechanistic and structural basis of Arc function is poorly understood. Arc has an N-terminal domain (NTD) involved in membrane binding and a C-terminal domain (CTD) that binds postsynaptic protein ligands. In addition, the NTD and CTD both function in Arc oligomerisation, including assembly of retrovirus-like capsids involved in intercellular signalling. To obtain new tools for studies on Arc structure and function, we produced and characterised six high-affinity anti-Arc nanobodies (Nb). The CTD of rat and human Arc were both crystallised in ternary complexes with two Nbs. One Nb bound deep into the stargazin-binding pocket of Arc CTD and suggested competitive binding with Arc ligand peptides. The crystallisation of the human Arc CTD in two different conformations, accompanied by SAXS data and molecular dynamics simulations, paints a dynamic picture of the mammalian Arc CTD. The collapsed conformation closely resembles *Drosophila* Arc in capsids, suggesting that we have trapped a capsid-like conformation of the human Arc CTD. Our data obtained with the help of anti-Arc Nbs suggest that structural dynamics of the CTD and dimerisation of the NTD may promote the formation of capsids. Taken together, the recombinant high-affinity anti-Arc Nbs are versatile tools that can be further developed for studying mammalian Arc structure and function, as well as mechanisms of Arc capsid formation, both *in vitro* and *in vivo*. For example, the Nbs could serve as a genetically encoded tools for inhibition of endogenous Arc interactions in the study of neuronal function and plasticity.

## Introduction

Activity-regulated cytoskeleton-associated protein (Arc) is a highly conserved protein in vertebrates that has emerged as a key regulator of long-term synaptic plasticity, with roles in postnatal cortical development, memory, and cognitive flexibility [1–3]. Arc is an immediate early gene in glutamatergic neurons that is highly expressed in a transient manner upon synaptic activation and salient behavioural experience [4–6]. Following transcription, Arc mRNA is transported from soma to dendrites, and translation occurs locally in or near dendritic spines of activated glutamatergic synapses [7]. The expression of Arc is dynamic, and the protein is quickly degraded [8–11]. Although loss-of-function studies have established causal roles for Arc [12–15], the molecular basis and cellular mechanisms of Arc are not fully understood [11,16]. Arc serves as protein interaction hub, with several binding partners in the postsynaptic membrane and dendritic spines of excitatory synapses, as well as in the neuronal nucleus [16]. Arc can also self-assemble, forming oligomers and large retroviral-like-capsids implicated in intercellular communication [17–20].

Arc is a fundamental regulator of synaptic plasticity, and high expression levels of Arc lead to the internalisation of AMPA-type glutamate receptors at the postsynaptic membrane [21,22]. Furthermore, Arc directly interacts with both endophilin, dynamin, and clathrin-adaptor protein 2 (AP2) [23,24]. Dynamin and endophilin both have essential late roles for clathrin-mediated synaptic receptor cycling, whereas AP2 has a critical role in initiating the process, as it coordinates cargo recruitment and selection by recruiting clathrin to the membrane [25]. A direct interaction of Arc with the AMPA receptor has been confirmed, as Arc binds the cytosolic tail of stargazin (Stg) [26,27]. Stg is an auxiliary subunit of AMPA receptors, associating with the receptor to modulate ion gating and receptor trafficking [28]. Long-term potentiation (LTP) is often induced *via* the activation of NMDA receptors, and Arc co-localises with NMDA receptors in postsynaptic complexes [7,29,30]. While Arc requires postsynaptic density protein 95 (PSD95) for localisation to the postsynaptic density, direct interactions with NMDA receptor subunits GluN2A and GluN2B have been identified [26,31]. GluN2A and GluN2B are vital for NMDA receptor-dependent LTP and long-term depression (LTD) [32,33]. Whether Arc regulates NMDA receptor function or serves as an integrator between LTP and LTD, is not yet fully understood.

Mammalian Arc (mArc) consists of two folded domains, a basic N-terminal domain (NTD) and an acidic C-terminal domain (CTD), connected by a flexible linker [19]. The linker region and the N- and C-termini are disordered. The CTD of Arc consists of the N-lobe and the C-lobe [26,34]. The solution structure of the rat Arc CTD revealed a rigid bilobar structure, where the two lobes are connected by a short non-helical region [31]. The N-lobe of the CTD harbours a peptide binding groove, which mediates many protein-protein interactions of Arc, including binding of Stg, GluN2A, GKAP and other peptides [26,31,34]. The binding pocket recognises a PxF/Y sequence motif, where the proline takes part in conserved C-H…*π* interactions, and the aromatic residue binds into a π-cluster within the hydrophobic core [34]. The N-terminal end of the CTD N-lobe is extended from the domain in the unbound state, but upon peptide binding, it folds against the bound peptide to form a β-sheet [31,34]. The C-lobe does apparently not participate in peptide binding, despite its structural similarity to the N-lobe [34].

The mArc NTD has low solubility, which has hindered detailed structural characterisation. It was suggested to form a coiled coil, and using small-angle X-ray scattering (SAXS) and Förster resonance energy transfer (FRET), it was shown to pack against the CTD, resulting in relatively compact full-length Arc [35]. The NTD is required for the association of Arc with endophilin [23], although direct binding has not been demonstrated. Full-length Arc, but not the CTD, associates with membranes rich in anionic lipids, suggesting a role for the NTD in membrane association [35]. Membrane association of Arc can introduce negative curvature in anionic membranes [36], and the NTD contains a cysteine cluster (^94^CLCRC^98^), which is S-palmitoylated *in vivo*, likely to facilitate membrane binding [37].

Transposable elements (TEs), such as retrotransposons, constitute a major component of vertebrate genomes that can cause deletions and genomic instability. However, depending on the genomic context at their insertion site, they can sometimes be positively selected for in an exaptation, or domestication, event. Arc, likely introduced into the tetrapod genome *via* retroviral insertion, was initially identified in a search for Gag homology proteins descendant from Ty3/Gypsy retrotransposons [38]. Structural studies on the Arc-CTD show striking similarity with capsid domains of retroviruses [26,35,39]. Arc retains its ancestral ability to form viral-like capsids, as it forms large capsids that encapsulate RNA to transfer it between neurons, or, as shown in *Drosophila*, from neuron to muscle [17,20]. Arc preferably forms these assemblies upon binding of its own mRNA [18]. In this way, Arc encapsulates its own mRNA and transports it between cells in a viral infection-like manner.

High-resolution structures of the capsids formed by *Drosophila melanogaster* Arc isoforms 1 and 2 (dArc1 and dArc2, respectively) revealed assemblies of icosahedral symmetry with 240 Arc protomers [40]. There are, however, fundamental differences in mArc and dArc. dArc lacks the coiled-coil NTD found in mArc, and the two likely originated from two separate domestication events, suggesting evolutionary convergence [20]. Both dArc isoforms spontaneously assemble into capsids at ionic strengths mimicking RNA binding *in vitro* [40]. This is thought to occur in part due to the inherent tendency of the dArc CTD lobes to oligomerise [41]. In contrast, the mArc CTD is monomeric in solution [31,34,35], but dimeric forms of the CTD appear to be important in capsid assembly [42]. mArc is unable to associate with mRNA and form higher-order oligomers in the absence of the NTD, and it has been suggested that full-length Arc is needed for the formation of the capsids, with the long linker region connecting the two domains facilitating domain swapping [18,35]. Alanine scanning identified a motif within the NTD, ^113^MHVWREV^119^, as a main facilitator of higher-order oligomerization and capsid formation in rat Arc (rArc). Furthermore, a crystal structure of short peptide harbouring the motif revealed a dimeric coiled coil. Upon replacement of the motif by poly-Ala, the full-length protein lost higher-order oligomerisation and is dimeric [18]. The structure of the mArc capsid and the mechanism of capsid formation are unknown.

In order to develop new tools for Arc structural and functional studies, we produced and characterised six high-affinity anti-Arc nanobodies (Nbs). Two of the Nbs facilitated crystallisation of the rat and human Arc-CTD, in an extended and collapsed conformation, visualising conformational dynamics of the Arc-CTD. The complementarity-determining region (CDR) 3 of NbArc-H11 bound deep into the peptide binding site of the Arc-CTD and inhibited peptide binding *in vitro*, suggesting applicability for studying Arc function at the molecular level. Our data suggest a mechanism of Arc capsid assembly, in which structural dynamics of the CTD facilitate capsomer formation, and NTD dimerisation leads to capsomer linking and formation of the mature capsid. The Arc Nbs provide an excellent starting point for further development of tools for both structural biology, imaging, immunodetection, and functional studies.

## Materials and methods

### Arc expression and purification

**Fig 1:**
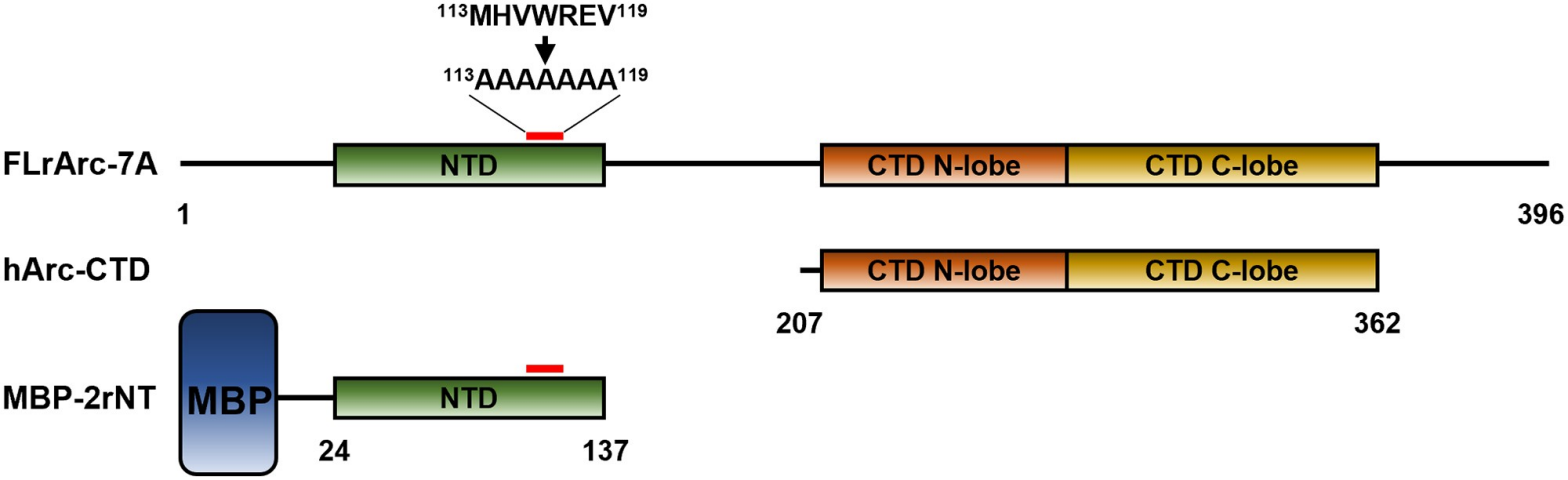
The Arc constructs used in this study. FLrArc-7A is full-length rArc (residues 1–397) with residues 113–119 mutated to Ala to hinder capsid formation [18]. hArc-CTD contains residues 207–362. Not shown is the N-terminal 6xHis-tag and the linker containing the TEV site. 2rNT is the NTD of rArc (residues 24–137) with the poly-Ala mutation. The domain was insoluble in the absence of its fusion partner, maltose binding protein (MBP). FLhArc corresponds to FLrArc-7A, but with the human Arc sequence without any mutations.

Fig 1 outlines the Arc constructs. FLrArc-7A, full-length rArc containing a poly-Ala mutation (residues 113–119), was in the pHMGWA vector [18], resulting in an N-terminal His_6_-maltose binding protein (MBP) fusion [43]. *E*. *coli* BL21(DE3)-RIPL cells were used for FLrArc-7A expression. The human Arc CTD (hArc-CTD) (residues 206–361) was expressed and purified as described [35]. The protein was expressed with an N-terminal His_6_ tag in *E*. *coli* BL21(DE3) cells using the pTH27 vector. The NTD of FLrArc-7A (2rNT) was expressed as an N-terminal His_6_-MBP fusion construct in pHMGWA using *E*. *coli* BL21(DE3)-RIPL cells. Details of recombinant Arc construct expression and purification are given in S1 Protocol.

### Anti-Arc nanobody generation

Anti-Arc Nbs were obtained commercially from NanoTag Technologies (Göttingen, Germany). Briefly, two alpacas were immunized a total of 6 times with the wild-type full length hArc and the FLrArc-7A mutant, gradually increasing the fraction of FLrArc-7A. After screening the obtained single-domain antibody (sdAb or nanobody) screening library using phage display and ELISA, sdAbs D4, C11, B5, H11, E5, and B12 were chosen as representatives of the four different sequence families and two unique binders and obtained as His_6_-TEV-Nb constructs in prokaryotic pNT1433 expression vectors.

For Nb production, BL21(DE3) competent *E*. *coli* cells were transformed with the expression clones. LB medium supplemented with 50 μg/mL kanamycin (10–20 mL) was inoculated with a single transformed colony, incubated overnight at 37°C and 170 rpm, diluted 100-fold into 0.5–2 L of the same medium and incubated at 37°C and 200 rpm. When OD600 reached 0.4–0.6, expression was induced via addition of 1 mM IPTG and maintained for 4 h at 30°C. Cells were harvested and pellets resuspended in 40 mM HEPES pH 7.5, 100 mM NaCl, 20 mM imidazole, 0.1 mg/mL hen egg white lysozyme. Cells were lysed via a single freeze-thaw cycle and sonication and soluble fraction harvested *via* centrifugation at 30000 g and 4°C for 30 min. NiNTA affinity purification and TEV proteolysis was carried out as above before a single negative NiNTA affinity purification step, concentration to 1–2 mL in 10 kDa MWCO spin concentrators and applied to either a HiLoad Superdex 75 pg 16/600 or a Superdex 75 Increase 10/300 GL SEC column (GE Healthcare, IL, USA) equilibrated in 20 mM Tris-HCl (pH 7.4) and 150 mM NaCl. Fractions deemed pure *via* SDS-PAGE were pooled, concentrated to 5–25 mg/mL, split into 50 μL aliquots, snap-frozen in liquid and stored at -80°C.

### Differential scanning fluorimetry (DSF)

To assess the thermal stabilisation of Arc constructs upon Nb binding, DSF was utilised. Proteins were diluted to 0.5–2 mg/mL in the assay buffer (20 mM Tris-HCl pH 7.4 and 150 mM NaCl) and mixed with 100x SYPRO-orange (in 50% (v/v) DMSO/assay buffer) in 384 well PCR plates to a final concentration of 5x, making the final DMSO concentration in the assay 2.5% (v/v). The final reaction volume was 18 μL. Fluorescence emission at 610 nm, following excitation at 465 nm, was measured in a LightCycler 480 LC RT-PCR system (Roche, Basel, Switzerland) over the temperature range 20–95°C with a temperature ramp rate of 2.4°C/min. Thermal denaturation midpoints (T_m_) were determined as the maximum of the first derivative of the melting curves.

### Analytical SEC analysis of Arc-Nb interaction

2.2 nmol of FLrArc-7A were mixed with an equimolar amount of Nb, incubated on ice for around 30 minutes before the sample (100 *μ*L) was injected on an Superdex 200 Increase 10/300 GL SEC column in 20 mM Tris, 150 mM NaCl (pH 7.5). 2.2 nmol of the unbound protein (apo) and Nbs alone were run as controls.

### Protein pulldown assays

For assessment of Nb binding and crude epitope mapping, protein pulldown assays were performed. 0.5 mg/mL of His-tagged hArc-CTD or His-MBP-tagged 2rNT were mixed with an equimolar amount of Nb and incubated on ice for 20–45 min. 200 μL of the solution were loaded on 100 μL of NiNTA agarose resin equilibrated in 20 mM Tris-HCl pH 7.4, 150 mM NaCl, 20 mM imidazole pH 7.5 and the mixture incubated at +4°C under gentle agitation for 1 h followed by centrifugation at 200 g and +4°C for 5 min. The supernatant (unbound protein) was removed, and the resin washed three times in the same buffer. To elute bound protein, the resin was incubated in the same buffer with 300 mM imidazole for 30 min before centrifugation as earlier. The fractions were analysed using SDS-PAGE.

### Isothermal titration calorimetry

The thermodynamics and affinity of Nb or peptide binding to FLrArc-7A were measured on a MicroCal iTC200 instrument (Malvern Panalytical, Malvern, UK). Binding of NbArcs to FLrArc-7A was measured in 20 mM Tris-HCl pH 7.4, 150 mM NaCl with 3.5–5 μM FLrArc-7A in the cell and 30–50 μM anti-Arc Nb in the syringe at 20°C and a reference power of 10–12 μcal/s, with a stirring speed of 1000 rpm. In all cases, except for NbArc-E5, a single 0.5-μL priming injection was followed by 19 × 2-μL 4 s injections of Nb with a spacing of 120 s and a filter period of 5 s. For NbArc-E5 binding to FLrArc-7A, the priming injection was followed by 38 × 1-μL injections. In the case of stargazin (Stg) binding to FLrArc-7A, 2 mM of the Stg peptide (RIPSYRYR with N-terminal acetylation and C-terminal amidation) dissolved in the assay buffer was injected (1 × 0.5 and 19 × 2 μL) into 198 μM FLrArc-7A. For the Stg-Nb displacement assay, 150 μM of NbArc-H11 was injected (1 × 0.5 and 19 × 2 μL) into 10 μM FLrArc-7A and 120 μM Stg. Data processing (peak integration and dilution heat subtraction) was carried out in the Origin Lab software (v. 2021). Binding enthalpy (ΔH), association constant (K_a_) and binding entropy (ΔS) were obtained by fitting the integrated thermograms with a 1:1 binding model.

### Small angle X-ray scattering

SAXS data from MBP-2rNT, and FLrArc-7A in the presence and absence of nanobodies, were collected on the SWING beamline of Soleil synchrotron, Saint Aubin, France in HPLC mode [44]. SAXS data from hArc-CTD in the absence and presence of Nbs were collected on the BM29 beamline of European Synchrotron Radiation Facility, Grenoble, France in HPLC mode [45,46]. Please see technical details of SAXS data collection in S2 Protocol.

Frame selection and buffer subtraction were carried out in CHROMIXS [47], primary analysis in PRIMUS [48] and distance distribution function analysis in GNOM [49]. *Ab initio* models were created using DAMMIN [50] and GASBOR [51]. Oligomer models were built from individual subunits based on SAXS data in CORAL [52]. Theoretical scattering curves of coordinate files were calculated using CRYSOL [53] and scattering-based normal mode analysis of crystal structures carried out in SREFLEX [54].

### hArc capsid preparation and Nb association assay

Full-length hArc (FLhArc) was cloned into the pETZZ_1a vector as a His-ZZ fusion protein construct with a TEV protease cleavage site [19]. *E*. *coli* BL21 CodonPlus cells transformed with the pETZZ_1a-FLhArc construct were used to inoculate 50 mL LB broth supplemented with 34 μg/mL chloramphenicol and 50 μg/mL kanamycin and incubated overnight at 28°C and 200 rpm. The starter cultures were diluted into 1 L LB broth with antibiotics, and incubated at 37°C and 200 rpm, until an OD_600_ of 0.6–0.8 was obtained. Expression was then induced by adding 0.5 mM IPTG and further incubated at 25°C and 200 rpm overnight. Cells were harvested at 6000 × *g* for 30 minutes and pellets stored at –20°C. Pellets were thawn on ice and resuspended in lysis buffer (5 mL/g pellet: 50 mM Na_2_HPO_4_/KH_2_PO_4_, 150 mM NaCl, 0.2% Tergitol^™^ solution (Merck, Darmstadt, Germany), and 2 mM DTT, pH 8.0, containing 1 tablet cOmplete^™^ with EDTA (Roche, Basel, Switzerland), 10 mM benzamidine and 0.2 mM PMSF) and homogenised using a Thomas pestle tissue grinder, before sonication on ice for 3 x 45 s at 20 W, with 45-s pauses. The soluble fraction was harvested by centrifugation at 14 000 × *g* at 4°C for 30 min. The soluble fraction was transferred to NiNTA agarose (Qiagen GmbH, Düsseldorf, Germany) equilibrated in 50 mM Na_2_HPO_4_/KH_2_PO_4_, 150 mM NaCl, and 2 mM DTT, pH 8.0, and incubated on rotation for 2 h at 4°C. The matrix was then washed with 50 mM Na_2_HPO_4_/KH_2_PO_4_, 150 mM NaCl, 0.2% Tergitol^™^ (Merck, Darmstadt, Germany), and 2 mM DTT, pH 8.0, until A_280_ = 0, before washing overnight with at least 30 bed volumes of 50 mM Na_2_HPO_4_/KH_2_PO_4_, 150 mM NaCl, and 2 mM DTT, pH 8.0. The matrix was then washed 10 bed volumes of 50 mM Na_2_HPO_4_/KH_2_PO_4_, 1 M NaCl, and 2 mM DTT, pH 8.0 followed by 10 bed volumes 50 mM Na_2_HPO_4_/KH_2_PO_4_, 150 mM NaCl, and 2 mM DTT, pH 8.0, before elution with the same buffer containing 300 mM imidazole. The protein was dialysed against 50 mM Na_2_HPO_4_/KH_2_PO_4_, 150 mM NaCl, and 0.5 mM TCEP, pH 7.4 to remove imidazole, before cleaving off the fusion protein with TEV protease. To remove the HisZZ-tag, the cutting reaction was added to Talon (Takara Bio Inc., Kusatsu, Shiga, Japan) and gently agitated for 1 h at 4°C, before elution and concentration at 2 000 × *g* at 4°C. Concentration was determined using the theoretical extinction coefficient (Abs 0.1%) 1.71. A 260/280 ratio >1 indicated nucleic acid content, and capsid formation was checked by negative-staining electron microscopy.

For negative-staining transmission electron microscopy, 300-mesh copper grids (#Cu-300, Electron Microscopy Sciences, Hatfield, PA, USA) with formvar (#15820, Electron Microscopy Sciences) and carbon coating (provided by MIC, Department of Biomedicine, University of Bergen) were glow discharged for 1 minute at 30 mA in a Dieno pico 100 vacuum chamber (Diener Electronics). 5 μL FLhArc from the Nb association assay were applied to grids and incubated for 2 min before excess sample was removed using Whatman^™^ filter paper. Grids were then washed two times in 20 μL water droplets before they were stained with 2% uranyl acetate for 1 min, with excess solution removed with filter paper in between each wash and after staining. FLhArc without Nbs were used as control. Micrographs were obtained with a JEOL JEM-1230 electron microscope operated at 80kV. Images were recorded at 80,000 × and 150,000 × magnification.

For SEC analysis, 2 nmol of FLhArc were incubated with 1.5-fold molar excess Nbs and then applied to a Superdex 200 Increase 10/300 GL column (GE healthcare) equilibrated in PBS buffer.

### Protein crystallisation and structure determination

Protein crystallisation was carried out in TTP3 sitting drop 96-well crystallisation plates (SPT Labtech, Melbourn, UK) using a Mosquito LCP crystallisation robot (SPT Labtech, Melbourn, UK). For crystallisation of Nb complexes, Nb was mixed with the target protein in 1.2–1.5 fold molar excess and the complex isolated via SEC on a Superdex 75 Increase 10/300 GL or Superdex 200 Increase 10/300 GL (GE Healthcare) column in 20 mM Tris-HCl pH 7.5, 150 mM NaCl. Fractions corresponding to the complex were pooled and concentrated to 10–37 mg/mL in 10 kDa MWCO spin concentrators and centrifuged at 20,000 g and 4°C for 5–10 min before concentration determination *via* absorbance at 280 nm and set up of 96-well screening plates with 270–600 nL drops with varying protein:reservoir ratios (2:1, 1:1 and 1:2), sitting over a 70 μL reservoir of the precipitant solution. Plates were incubated at either 8°C or 20°C.

Diffraction data were collected on beamlines I03 (Diamond Light Source), P14 (EMBL/DESY), and P11 (DESY) [55]. For experimental details of individual crystal growth, diffraction data collection, and structure solution, please refer to S3 Protocol.

Diffraction data were processed in XDS [56]. Analysis of data quality was carried out in XTRIAGE [57] and phases solved using molecular replacement (MR) in PHASER [58]. Refinement was carried out in PHENIX.REFINE [57] and manual model building in Coot [59]. Anisotropy analysis and anisotropic scaling were carried out using the STARANISO and the UCLA-DOE lab—Diffraction anisotropy [60] web servers. Structure validation was performed using MolProbity [61]. Analysis of Nb-epitope interfaces, symmetry-based oligomers, and interacting residues was carried out using PDBsum [62] and PISA [63]. Figures of crystal structures were prepared in PyMOL (Schrödinger, LLC). Crystal diffraction and refinement statistics are shown in S1 Table. The refined coordinates and structure factors were deposited at the Protein Data Bank with entry codes 7R20 (Nb E5), 7R24 (rArc-CTD complex), 7R23 (extended hArc-CTD complex), and 7R1Z (collapsed hArc-CTD complex).

### Molecular dynamics simulations

Atomistic MD simulations were carried out on the hArc-CTD in GROMACS [64]. The hArc CTD crystal structure was stripped of Nbs, protons and ions, and assigned the predicted protonation state at pH 7.0 using PROPKA in PDB2PQR [65]. Protonated hArc-CTD was placed in a cubic box with a 10 Å extension around the protein. Solvation was done with the TIP3P water model in 0.15 M NaCl. The models were subjected to conjugate gradient energy minimisation with a steepest decent step every 50th step and a maximum of 5000 steps. Temperature (NVT) equilibration to 300 K and pressure (NPT) equilibration, via isotropic pressure coupling, were carried out using the Berendsen thermostat. MD simulations were carried out using the OPLS-AA/L force field [66] and leap-frog algorithm, while retaining constant temperature and pressure using a velocity-rescale thermostat and a Parinello-Rahman barostat, respectively. The MD trajectories were uploaded to Zenodo [67–70].

### Dynamic light scattering

Dynamic light scattering (DLS) of MBP-2rNT was measured on a Zetasizer Nano ZS DLS instrument (Malvern Panalytical) on 1 mg/mL sample in a 3x3 mm quartz cuvette (Hellma analytical) at 25°C. Prior to the measurement, samples were filtered through 0.22 μm centrifugal filters at 6,000 g and 4°C for 8 min. 30 μL of sample were loaded in the cuvette, allowed to equilibrate for 120 s and measured three times, with each run consisting of 10 accumulative measurements. Hydration radius (R_H_) was determined as the maximum of the volume fraction size distribution profile.

### Solubilisation of the Arc NTD in L-Arg

As the NTD of Arc had previously been shown to be highly insoluble in the absence of its MBP fusion partner, solubilisation in L-arginine (L-Arg) was attempted. Purified MBP-2rNT fusion, at ~1 mg/mL, was dialysed against 1 L of 20 mM HEPES pH 7.5, 150 mM NaCl and 375 mM L-Arg pH 7.5. Following ~3 hr dialysis, the sample was supplemented with ~500 μg TEV protease and further dialysed against the same buffer overnight. For removal of the cleaved His-MBP affinity tag, the protein was subjected to negative NiNTA affinity purification and the unbound protein further purified on amylose resin to remove residual MBP. Unbound protein from the amylose resin was concentrated to 500 μL and injected on a Superdex 75 increase 10/300 GL column equilibrated in 20 mM HEPES pH 7.5, 150 mM NaCl and 375 mM L-Arg pH 7.5. Purity of the eluted NTD peak was assessed through SDS-PAGE. Although soluble NTD could be obtained through this method, the low yield did not allow for further biophysical characterisation.

## Results

### Anti-Arc nanobodies and their interaction with FLrArc-7A

**Fig 2:**
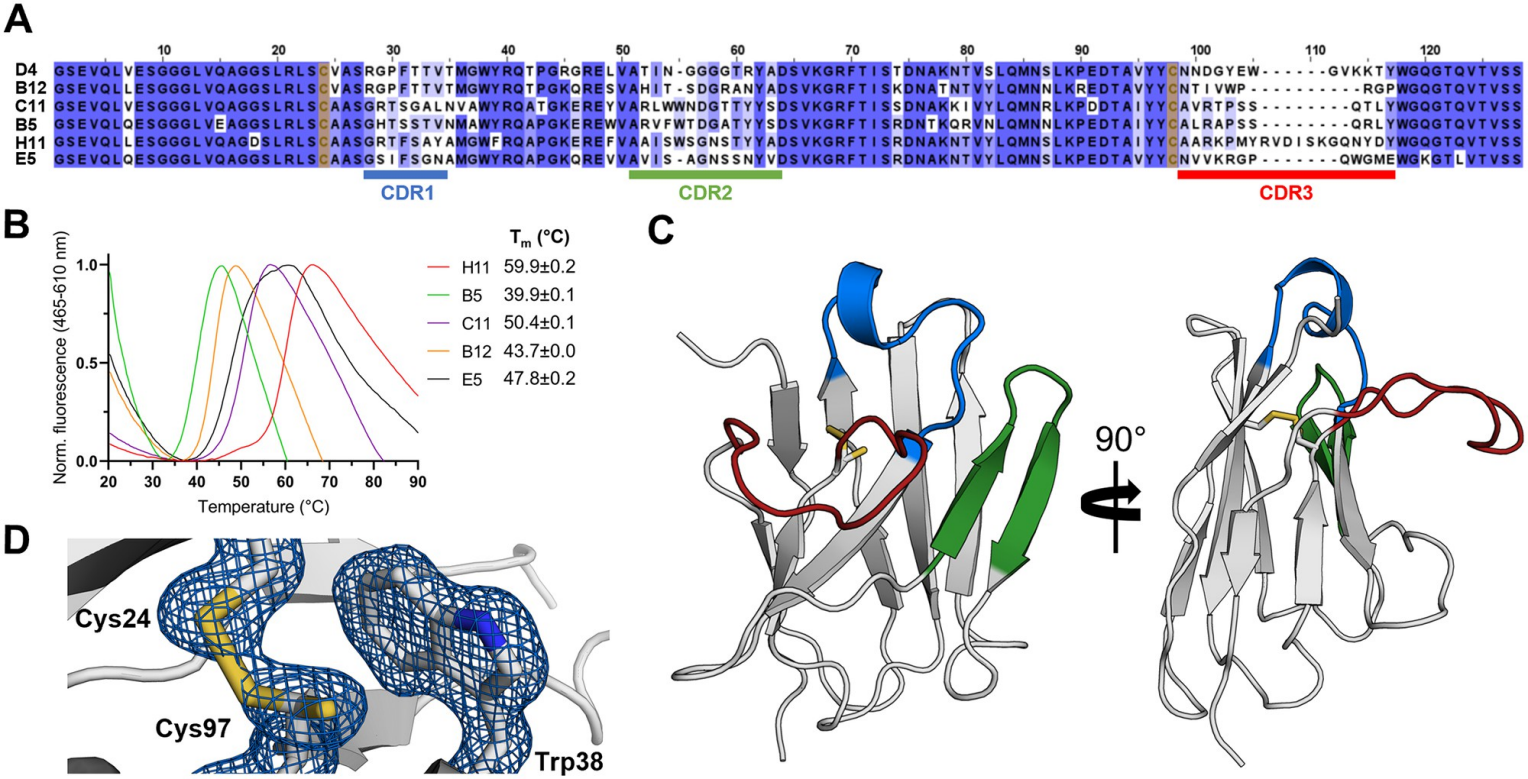
Anti-Arc nanobodies. **A** Sequence alignment reveals the location of the three CDRs, highlighting the long CDR3 of NbArc-H11. Sequences are coloured by conservation, and Cys24 and Cys92 (Kabat numbering) are highlighted in yellow. The alignment was produced using Clustal Omega [71] and JalView [72]. **B** Determination of NbArc T_m_ using DSF (N = 3). The T_m_ of NbArc-D4 could not be determined. **C** 1.42-Å crystal structure of NbArc-E5. Colouring of CDRs is the same as in panel *A*. **D** The conserved central disulphide bond, formed by oxidation Cys24 and Cys97, packs against Trp38 and is partially reduced. Electron density is shown as a blue mesh at 1.5σ.

To aid in structural and functional studies on Arc, Nbs were raised against the dimeric poly-Ala rArc mutant (FLrArc-7A). The immunisation resulted in 92 positive Nb clones, and Nbs D4, H11, B5, C11, B12 and E5 were chosen for further characterisation, as they represented different sequence families within the collection of clones. The sequence alignment (Fig 2A) highlights the variable CDR loops and an extended CDR3 in NbArc-H11. DSF was used to estimate thermal stability of the Nbs (Fig 2B). No sequence-specific trend could be identified, but as the framework region (FR) is highly conserved, the varying thermal stability likely depended upon paratope loops, mainly CDR3. NbArc-H11 showed the highest T_m_ despite having the longest CDR3, suggesting some folding of the CDR3 or its interaction with the FR surface.

To further characterise the Nbs in their free state, Nb E5 was crystallised, allowing for structure determination at 1.42-Å resolution (Fig 2C). The structure shows the typical immunoglobulin fold, an incomplete β-barrel of nine anti-parallel β-strands. The overall structure is compact, and the three CDR loops extend from the protein. Despite its elongated nature, the CDR3 loop is rigid in relation to the rest of the protein (S1A Fig). Hydrophobic residues of the CDR3 pack onto the exterior of the central β-barrel to shield non-polar side chains (S1B Fig), which might account for the high solubility of this Nb. Furthermore, the two tryptophans and the basic residues in the CDR3 loop suggest a negatively charged, buried epitope. The central disulphide (Cys24-Cys97) appeared mostly reduced, possibly due to radiation damage. Upon further inspection, it became clear that it was partially oxidised, and was refined to 20 and 80% occupancies for the oxidised and reduced rotamers of Cys97, respectively (Fig 2D).

### High-affinity Nb binding to the CTD of Arc results in thermal stabilisation

**Fig 3:**
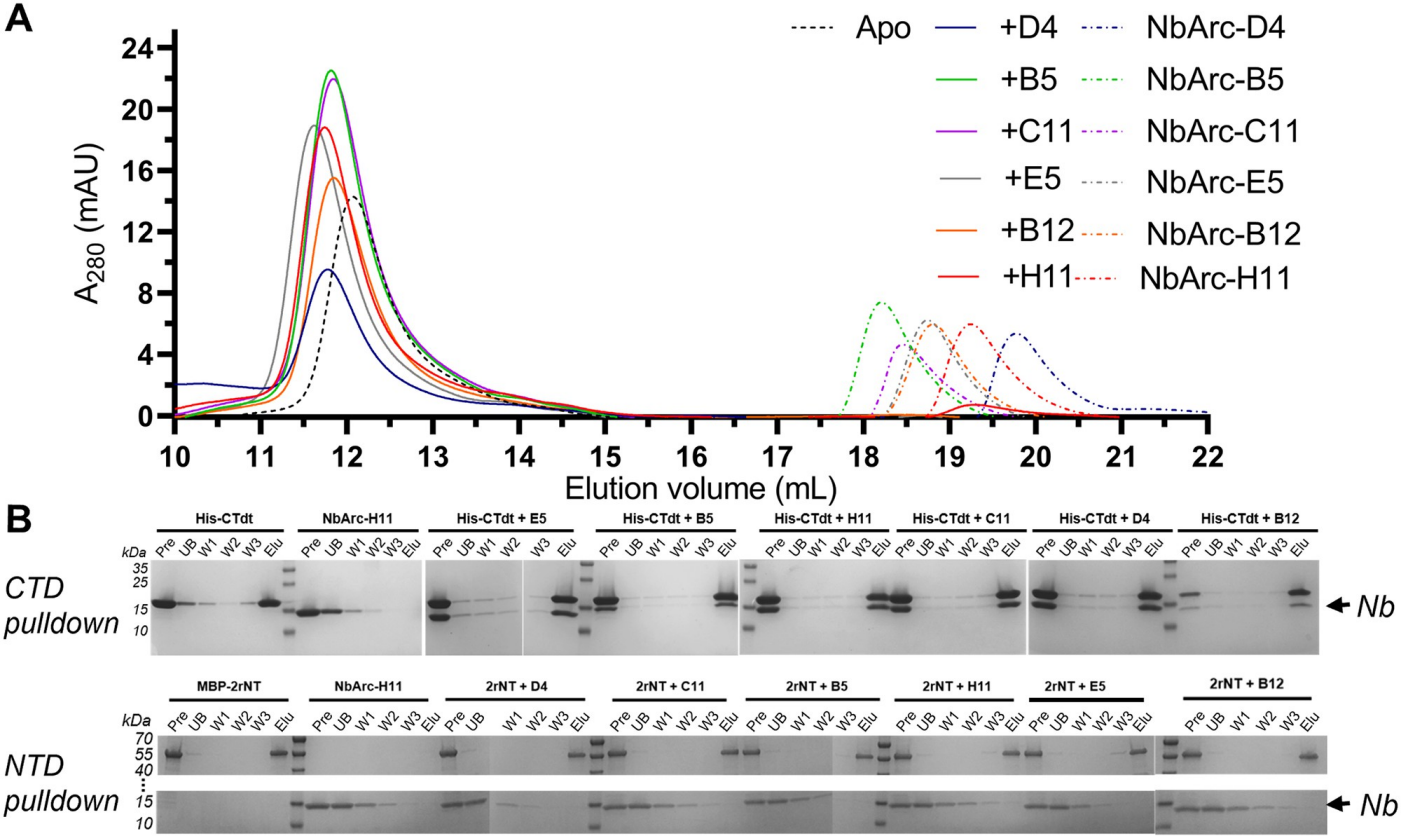
Initial characterisation of Nb binding to Arc. **A** Analytical SEC demonstrated Nb binding to FLrArc-7A. **B** NiNTA Nb pulldown assay with equimolar amounts of His-tagged hArc-CTD (CTD) or MBP-2rNT (NTD) and untagged Nbs, followed by SDS-PAGE analysis. All Nbs associated with the CTD (upper panel), as noted by the presence of the Nb band in all complex elutions, whereas none bound to the NTD (lower panel). Pre denotes the complex prior to loading on the matrix, UB the unbound fraction, W1-3 the three washing steps, and Elu the eluted fraction. All unmodified SDS-PAGE gel images of the current study are in S14 Fig.

To confirm the activity of the purified anti-Arc Nbs and crudely map their epitopes, analytical SEC and protein pulldown experiments were performed (Fig 3). Analytical SEC was chosen to quickly confirm binding to FLrArc-7A. The results (Fig 3A) confirmed that all the purified Nbs bound Arc, as an increase was observed in the elution volume of the complexes when compared to the unbound protein alone. Although accurate molar mass and stoichiometry could not be determined from this experiment, the small shift in elution volume proposed the overall fold and the oligomeric state of the FLrArc-7A to remain unaltered upon Nb binding. To further map the binding sites, Arc constructs were subjected NiNTA protein pulldowns with the Nbs (Fig 3B). All the Nbs associated only with the CTD, but none bound to MBP-2rNT. This suggests that the NTD might have been inaccessible upon immunisation, possibly due to its position at the dimer interface.

**Fig 4:**
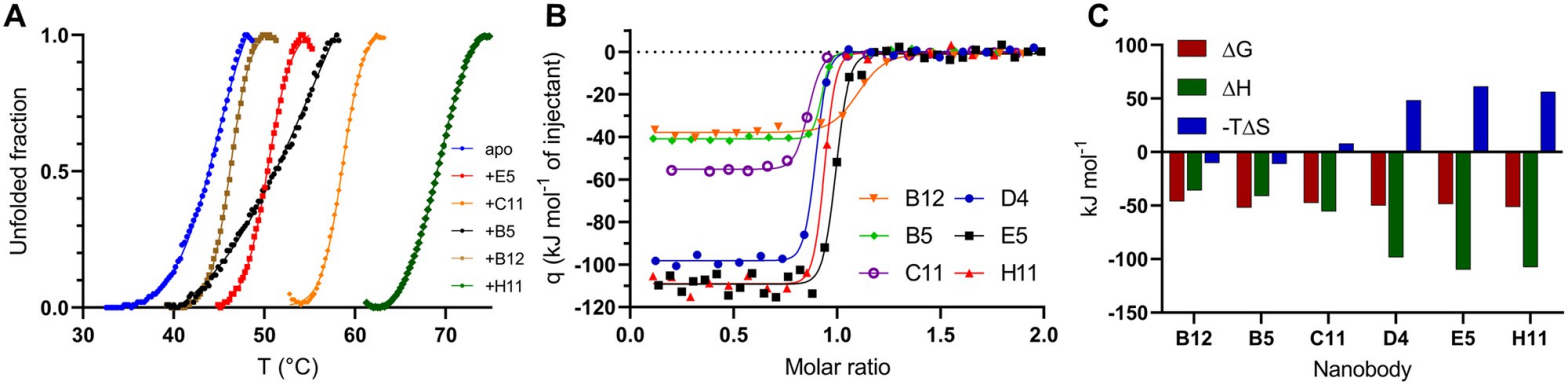
Nb binding to FLrArc-7A. **A** DSF melting curves of apo FLrArc-7A and in complex with Nb. Portions of the curves, other than the unfolding transition, are not shown for clarity. T_m_ values are shown in Table 1. **B** ITC titrations of Nb into FLrArc-7A. Raw ITC thermograms can be seen in S2 Fig. **C** Contributions of ΔH and ΔS to the ΔG of Nb binding, highlighting enthalpy-entropy compensation.

To study the thermal stabilisation of FLrArc-7A upon Nb binding, DSF was utilised (Fig 4A, Table 1). Binding of NbArc-C11 and -H11 resulted in highest stabilisation. The large increase in T_m_ suggested that these Nbs either stabilised flexible regions of Arc or selected for a conformer much more stable than free Arc. As all unfolding transitions observed for the Nb-Arc complexes were cooperative, and the measured T_m_ was always higher than that of the individual components, the observed transition corresponded to the unfolding of the complex, except perhaps in the case of the rArc+B5 complex. Assessing the unfolding of the rArc+D4 complex proved unsuccessful, as two obscure transitions were observed, indicating complex dissociation prior to denaturation.

**Table 1:** Thermal stability and thermodynamics of Nb binding to FLrArc-7A.

Nb	ΔT_m_ (°C)	Number of sites	*K*_*d*_ (nM)	ΔH (kJ mol^-1^)	-TΔS (kJ mol^-1^)	ΔG (kJ mol^-1^)
B12	+1.1	1.040±0.010	7.34±2.97	-36.00±0.70	-10.11	-46.11
B5	+8.7	0.875±0.003	0.66±0.29	-41.05±0.29	-10.99	-52.04
C11	+13.4	0.815±0.002	3.49±0.85	-55.62±0.43	7.97	-47.64
D4	N/D	0.845±0.003	1.19±0.27	-98.49±0.64	48.37	-50.12
E5	+5.2	0.967±0.003	2.34±0.63	-110.00±1.00	61.27	-48.73
H11	+24.0	0.892±0.003	1.12±0.45	-107.57±0.51	56.28	-51.29

ΔT_m_ was measured using DSF (Fig 4A). Thermodynamics of binding were derived from ITC (Fig 4B). N/D: Not determined.

ITC was used to further assess the binding of the Nbs to FLrArc-7A (Fig 4B and 4C, Table 1). All individual Nbs bound to Arc in 1:1 stoichiometry, in an enthalpy-driven manner. All bound with similar ΔG of around -50 kJ mol^-1^, with estimated *K*_*d*_ of 0.6–7.3 nM (Table 1). However, it should be noted that ITC is generally limited to affinities in the *K*_*d*_ range of 10 nM– 100 μM [73]. The contributions of the enthalpy and entropy terms differed considerably (Fig 4C). For NbArc-B12, -B5 and -C11, the entropy term was negligible, and the interaction was almost entirely enthalpy driven. NbArc-D4, -E5 and -H11 showed unfavourable binding entropy, compensated for by a large favourable ΔH. For B12, B5 and C11, the negligible binding entropy suggested no considerable rigidification in the complex. However, the highly unfavourable binding entropy of D4, E5 and H11 suggested more rigidification and increased bonding (hydrogen bonds and salt bridges). Altogether, ITC showed that the Nbs bind to FLrArc-7A with high affinity, with varying modes of enthalpy-entropy compensation, reflecting different epitopes.

### SAXS analysis of FLrArc-7A Nb complexes

**Fig 5:**
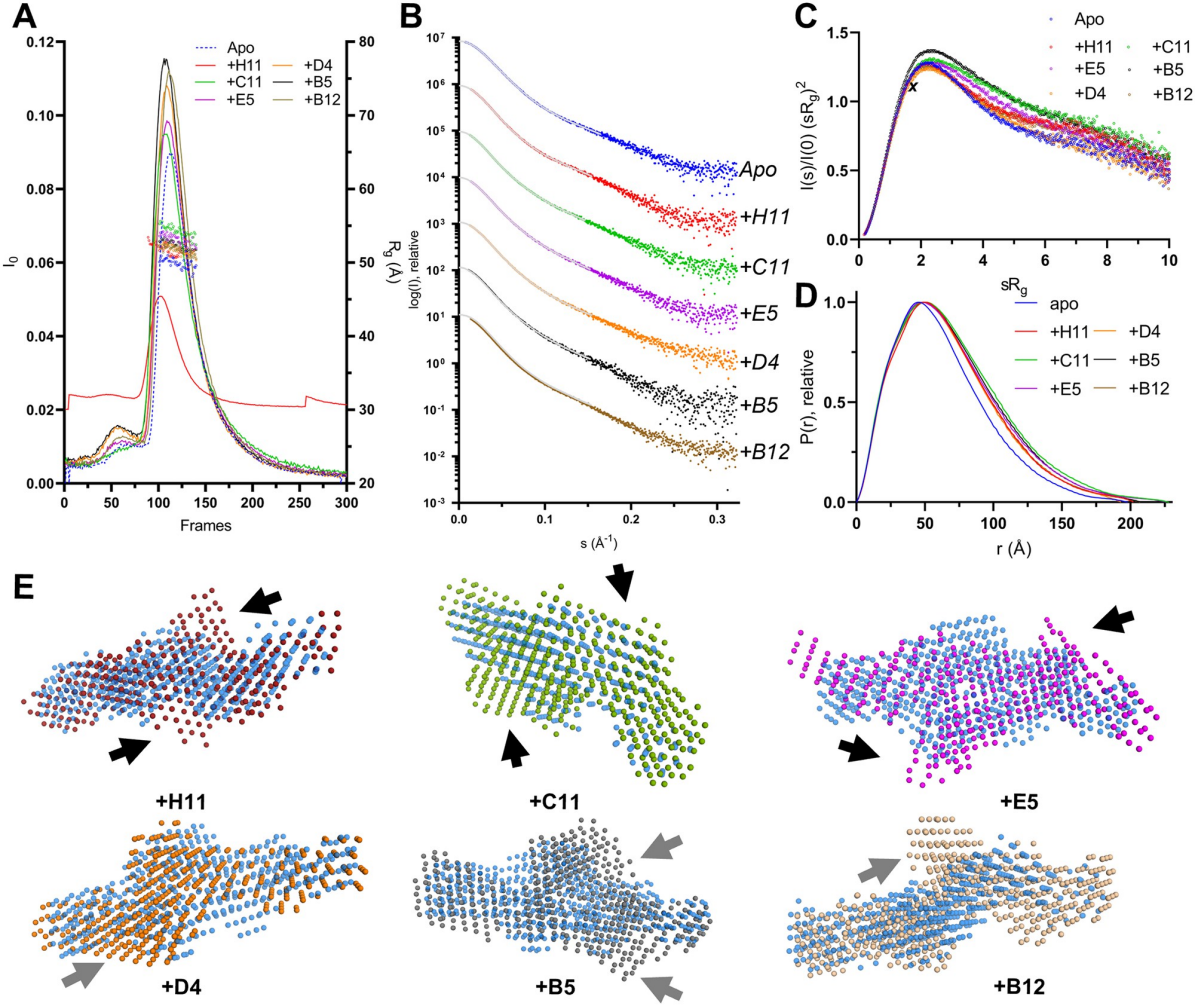
SEC-SAXS analysis of FLrArc-7A Nb complexes. **A** SEC-SAXS profiles of the complexes and unbound (Apo) FLrArc-7A. The estimated R_g_ of the frames used for processing is shown in open circles. All complexes were measured in 20 mM HEPES, 150 mM NaCl, 0.5 mM TCEP, pH 7.5. rArc+H11 was measured on a separate occasion in 20 mM Tris, 150 mM NaCl, pH 7.4, which caused differences in intensity and the elution volume of the main peak. **B** Scattering curves of rArc and rArc-Nb complexes, offset for easier visualisation. Data fits from GNOM are shown as grey lines. **C** Dimensionless Kratky plot. The maximum of an ideal rigid spherical particle ($\sqrt{3}$, 1.104) is marked by ***X***. **D** Distance distribution profiles. **E** *Ab initio* models of Nb-bound FLrArc-7A in various orientations, aligned with the model of the unbound protein (in transparent blue). Arrows indicate additional volumes in the complex models, which might correspond to bound Nb. Black arrows indicate two-fold symmetric binding, and grey arrows indicate additional volumes following no specific symmetry. Models were produced in DAMMIN with no forced symmetry, with *χ*^2^ values of 0.992 (apo), 1.063 (+H11), 1.090 (+C11), 1.106 (+E5), 1.287 (+D4), 1.090 (+B5) and 1.090 (+B12).

SAXS was used to estimate the solution structure of the Nb complexes of FLrArc-7A, and to detect conformational changes in Arc upon Nb binding (Fig 5, Table 2). To measure scattering from only complexes and not unbound FLrArc-7A or Nbs, the complexes were separated from unbound FLrArc-7A using SEC and SAXS frames collected as the proteins eluted. The data indicate that in all cases, the Nb bound to the FLrArc-7A dimer in 2:2 stoichiometry, confirming the Arc:Nb stoichiometry of 1:1 from ITC. The Kratky plot (Fig 5C) indicated varying rigidity of the complexes, without significant rigidification upon binding. The distance distributions (Fig 5D) differed in all cases from that of unbound FLrArc-7A, showing an expansion in structure. R_g_ increased in all cases, accompanied by small changes in D_max_ (Table 2). The highest increase in R_g_, accompanied with an almost unchanged D_max_, was observed in complex with C11. This indicates binding further from the centre of mass of FLrArc-7A. An increase in D_max_ was observed for E5 and H11, which indicated binding to the end of the long axis of the Arc dimer; the epitope for these Nbs must lie at the ends of the elongated dimer, further allowing to map domain location in the Arc dimer.

**Table 2:** Parameters derived from SAXS of FLrArc-7A Nb complexes.

Complex	Apo	+B5	+B12	+C11	+D4	+E5	+H11
R_g_ (Å)	48.16	51.97	51.67	54.18	51.39	52.38	51.48
Porod volume (nm^3^)	256	301	305	316	293	303	324
P(r) R_g_ (Å)	50.57	54.75	53.62	56.63	53.80	56.03	55.47
D_max_ (Å)	198.0	198.9	193.5	206.8	203.5	227.5	229.0
Quality est. GNOM	0.74	0.79	0.81	0.78	0.79	0.76	0.77
Q_p_ Mw (kDA)	135.4	152.9	156.5	164.4	167.1	160.2	156.1
1:1 complex mass (kDA)	45.0	58.2	57.7	58.1	57.9	57.8	59.1
Oligomeric state/stoichiometry	Dimer	2:2	2:2	2:2	2:2	2:2	2:2

R_g_ is the radius of gyration, D_max_ the longest dimension of the particle, and Q_P_ Mw the molar mass estimate produced using the Porod invariant, Q_P_.

*Ab initio* dummy atom models are compared in Fig 5E. Changes were observed in the calculated molecular envelope, and with NbArc-H11, -C11 and -E5 bound, additional volumes indicated the presence Nbs bound to each monomer. The changes in the overall shape of the protein may be related to reduced fluctuations in the disordered regions of FLrArc-7A upon Nb binding. The SAXS experiments demonstrated that all Nbs bound the FLrArc-7A dimer in 2:2 stoichiometry, but the low-resolution nature of SAXS and the low molar mass of the Nbs compared to dimeric Arc did not allow for accurate epitope mapping.

### Anti-Arc nanobodies associate with high-molecular-weight hArc without promoting disassembly

**Fig 6:**
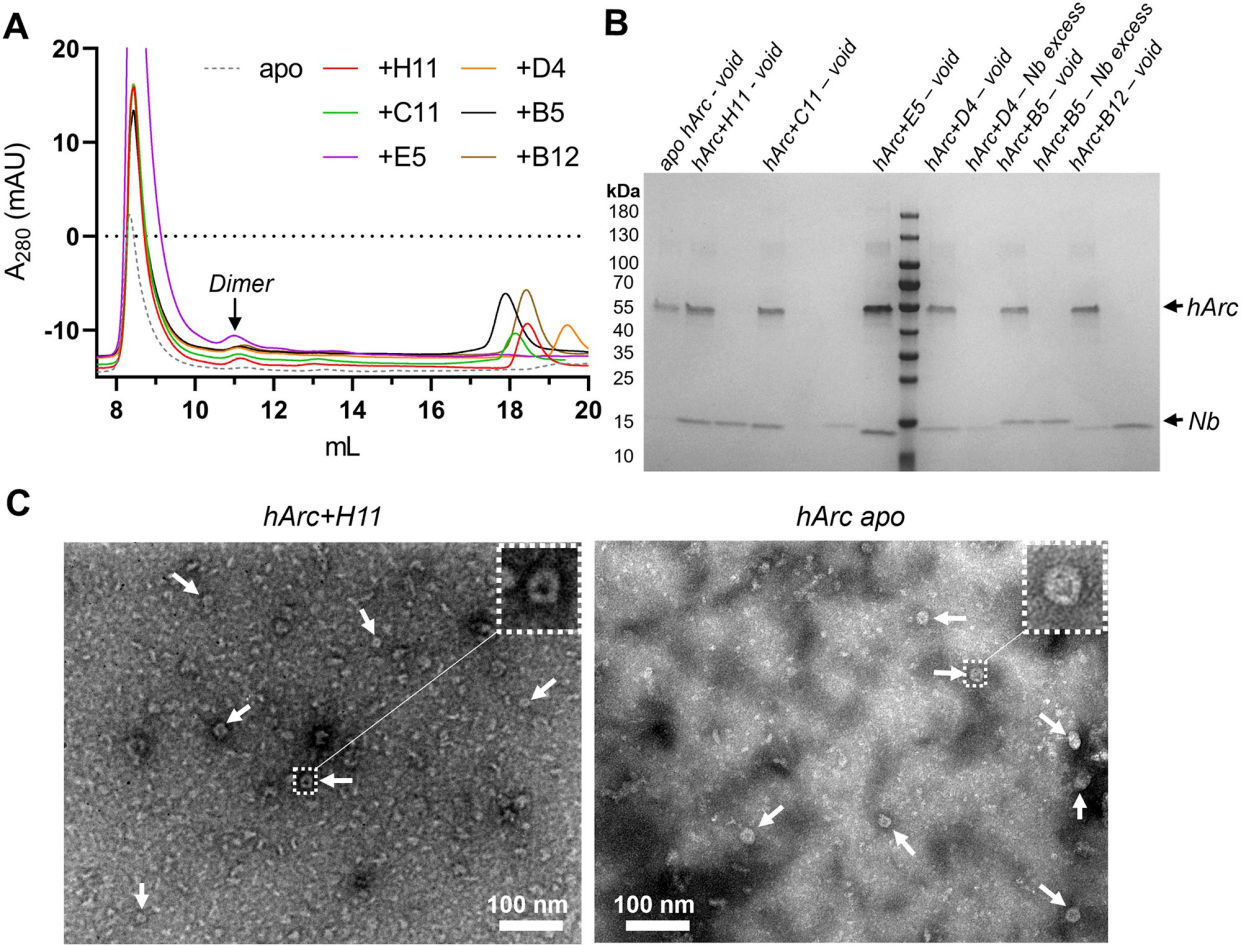
Rudimental analysis of Nb association with hArc capsid fraction. **A** Analytical SEC of capsid hArc-Nb complexes. 2 nmol of Arc capsids were mixed with a 1.5-fold molar excess of Nbs and run on a Superdex 200 Increase 10/300 GL in PBS buffer. Noted is the presumably dimeric peak present in some Nb complexes, with an elution volume corresponding to that of the FLrArc-7A complexes. NbArc-E5 was accidentally mixed with 4 nmol of hArc, resulting in the large void peak and lack of Nb excess peak. **B** SDS-PAGE analysis of SEC fractions, demonstrating that Nbs co-elute with the capsids. **C** Negative staining TEM images of capsid hArc at 150,000x magnification with NbArc-H11 (left) and without Nb (right). Arrows point at capsids, and a magnified view of selected capsids is shown.

Considering the reported ability of some Nbs raised against viral proteins to associate with capsids and facilitate deactivation [74,75], we investigated whether the same applied for the anti-Arc Nbs. The association of the Nbs to hArc capsid-containing fractions was assessed using analytical SEC (Fig 6). Although a minor peak of a similar elution volume as Nb-bound, dimeric FLrArc-7A, appeared upon addition of the Nbs, most co-eluted with the high-molecular-weight forms of hArc in the void volume (Fig 6B). Furthermore, virus-like capsid structures, like those present in hArc without Nbs, were apparent in negative staining TEM images in the presence of Nb H11 (Fig 6C and 6D). The dimer peak, not present in the absence of Nbs, was most prominent with H11, suggesting some degree of Nb-induced capsid dissociation. The Nbs could be associated with the capsids, or other high-MW forms, but did not induce conformational changes that would directly lead to capsid disassembly.

### Crystal structure of an rArc-CTD ternary nanobody complex

We attempted the crystallisation of the FLrArc-7A mutant, using the six anti-Arc Nbs as crystallisation chaperones. However, despite extensive efforts, none of the complexes with one Nb grew crystals of sufficient quality for diffraction data collection. Therefore, crystallisation with more than one Nb was attempted. Analytical SEC showed the E5+C11, H11+C11, H11+B5 and E5+B5 Nb pairs to be compatible, whereas in other cases, binding of the second Nb caused dissociation of the first (S3 Fig). Crystallisation of FLrArc-7A was attempted in complex with both E5/C11 and H11/C11, as these individually led to the most thermal stabilisation (Fig 4, Table 1). FLrArc-7A in complex with H11 and C11 showed crystal growth after extended incubation. The FLrArc-7A+H11+C11 crystals diffracted to 2.7-Å resolution, but the asymmetric unit was too small to contain full-length Arc and both Nbs. Indeed, the crystals contained the rArc CTD in complex with both Nbs, suggesting to proteolytic cleavage of the linker region.

**Fig 7:**
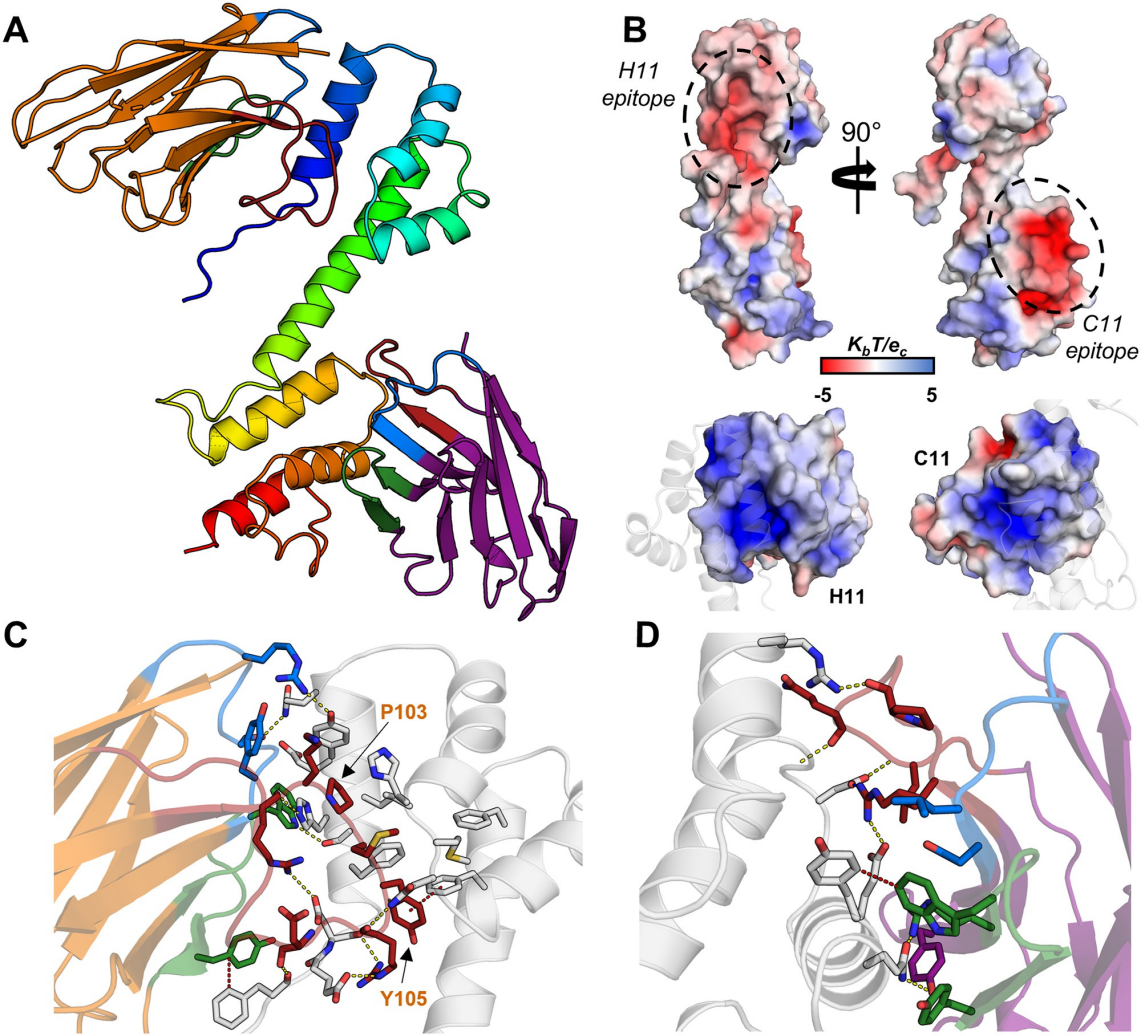
The crystal structure of rArc-CTD in complex with NbArc-H11 and -C11. **A** The overall structure. The CTD of rArc is shown in blue to red from the N- to the C-terminus. NbArc-H11 is shown in orange and NbArc-C11 in purple. CDR 1, 2, and 3 are coloured blue, green, and red, respectively. **B** Both Nbs bind acidic surface patches of the CTD (above); the surface of the Nb CDRs is positive (below). Electrostatic potential was calculated using ABPS [76]. The epitopes correspond to the ligand peptide binding site of the N-lobe (H11) and the C-lobe oligomerisation surface (C11). **C** Pro103 and Tyr105 of H11 extend into the Arc N-lobe hydrophobic pocket. **D** C11 binding of the rArc-CTD. Polar contacts (yellow) and π-π interactions (red) are indicated with dashes.

Residues 211–356 of rArc, corresponding to the whole Arc-CTD, could be built, while the NTD was not detected in electron density. Nbs H11 and C11 could be fully built, apart from the first two N-terminal and the last C-terminal residues (Fig 7). The ternary complex contains the rArc CTD, *i*.*e*. the N-lobe, a 4-helix orthogonal bundle, and the C-lobe, a 5-helix orthogonal bundle, connected by a rigid central *α*-helix (Fig 7A). Nbs H11 and C11 bound to distinct, acidic epitopes on the N-lobe and the C-lobe, respectively. Accordingly, the electrostatic surface of the CDRs of both Nbs is basic (Fig 7B). A large majority of crystal contacts were formed exclusively by the Nbs (panel A in S4 Fig).

The elongated CDR3 loop of H11 protrudes from the β-barrel fold and extends into the N-lobe of the CTD. The binding buries a large area on the CTD, 1044 Å^2^, and the first helix of the N-lobe is engulfed in a crevice between CDR loops 1 and 3 of H11 (Fig 7C). CDR3 accounts for most contacts, and Pro103 and Tyr105 of CDR3 show CH…π and π-π interactions with aromatic residues within the pocket of the N-lobe. This groove of the N-lobe is the ligand peptide binding site of Arc [26,31,34], indicating that Nb H11 could be used to modulate protein-protein interactions of the Arc N-lobe. Additionally, H11 showed interactions with the N-terminal tail of the N-lobe, suggesting that it might affect the conformation of the long linker connecting the NTD and CTD in full-length Arc.

NbArc-C11 bound a solvent-exposed epitope in the C-lobe, burying 738 Å^2^ of Arc surface (Fig 7D). The interacting area is typical for antibody-antigen interactions [77]. The binding site overlaps with the suggested oligomerisation surface of the C-lobe [42], indicating that C11 could be used to modify Arc interactions and oligomeric state.

### Crystallisation of human Arc CTD

Limited structural information has been available on hArc, apart from the individual N- and C-lobes [34]. Taking advantage of NbArc-H11 and -C11, crystallisation of hArc-CTD was carried out. The structure of the hArc-Nb ternary complex was refined to 2.77-Å resolution and contained the CTD of hArc (residues 209–355) in complex with both Nbs (panel A in S5 Fig). The two lobe domains are almost identical to rArc (panel B in S5 Fig), with aligned C*α*-RMSD of 0.65 Å for both lobes. However, the central helix connecting the two lobes breaks in hArc at residues 275–277, resulting in an altered relative orientation of the lobes. Compared to rArc, the hArc complex had altered crystal packing, and the Nbs play an even larger role in crystal contacts (panel B in S4 Fig).

The binding mode of H11 to hArc was similar to that observed in the rArc structure, burying 1073 Å^2^ on the CTD. However, due to the bending of the central helix in hArc-CTD, the binding mode by C11 was slightly altered, and the buried surface area of hArc was increased to 848 Å^2^. This was achieved by additional interactions of C11 with both the central helix and the N-lobe, suggesting that C11 might prefer a slightly bent conformer of the CTD by interacting with both lobes.

### NbArc-H11 inhibits Arc ligand peptide binding

**Fig 8:**
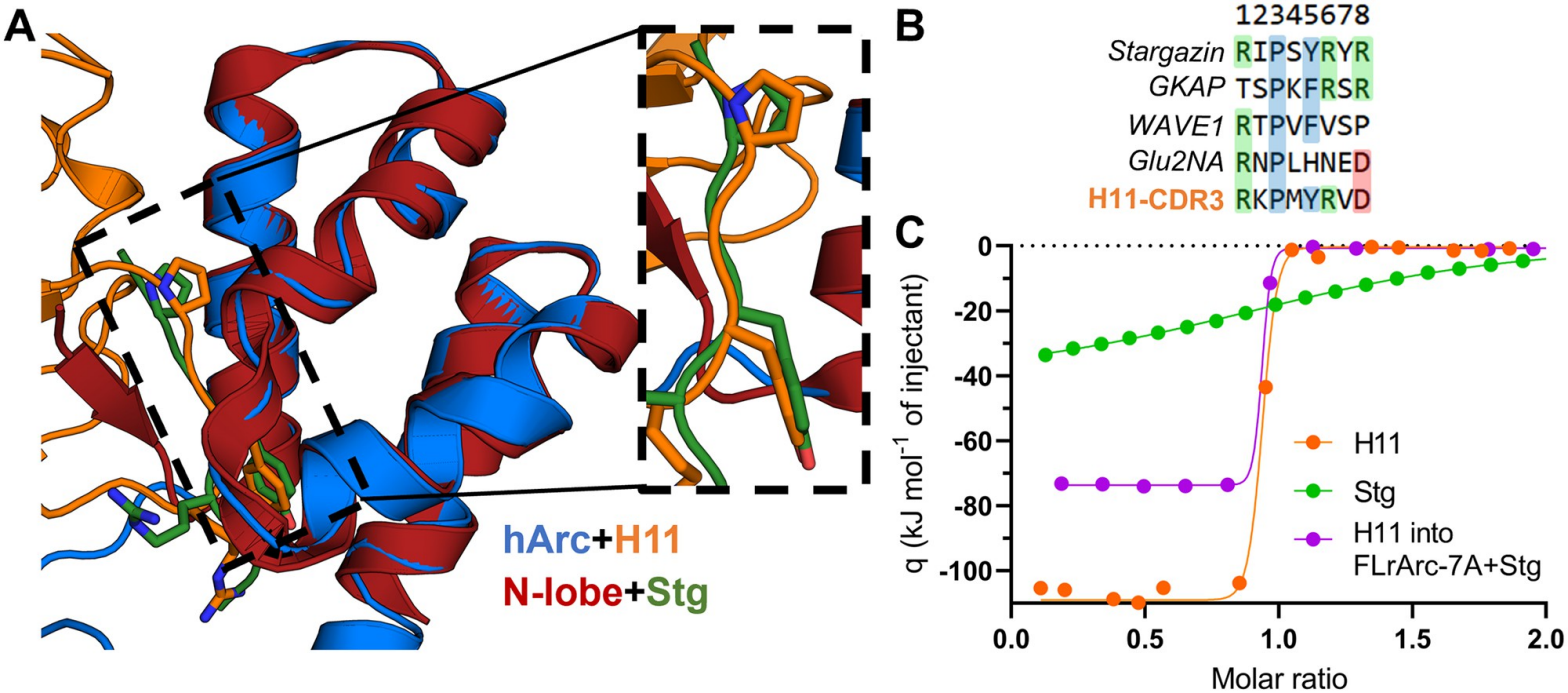
NbArc-H11 as a peptide binding inhibitor. **A** Alignment of hArc-CTD bound to H11 with the crystal structure of the human N-lobe with bound Stg (6TNP, [34]) reveals the identical mode of binding. **B** Sequences of various Arc ligand peptides aligned with the sequence of the H11-CDR3. **C** ITC displacement assay. Shown are the integrated thermograms of H11 and Stg titrated into FLrArc-7A, and H11 titrated into FLrArc-7A bound to Stg. The decreased binding enthalpy of H11 binding to Stg bound FLrArc-7A suggests that H11 binding leads to Stg dissociation. Raw thermograms are shown in S6 Fig. The H11 titration curve is the same as shown in Fig 4B.

Arc localises to the postsynaptic density (PSD) and binds ligand peptides in a hydrophobic groove located in N-lobe of the CTD. These peptides, from the PSD and dendritic spine, include Stg, GKAP, GluN2A, and WAVE1, involved in regulation of structural and functional synaptic plasticity [26,31,34]. The CDR3 loop of Nb H11 bound to the same site as the ligand peptides [26,34] in the crystal structures, and indeed, the consensus ligand sequence motif P-x-Y is present in the H11-CDR3 loop, resulting in a nearly identical mode of binding (Fig 8A and 8B). This suggested that H11, which binds with nM affinity, could be used to efficiently displace bound peptides, which have affinities of 20–500 *μ*M [34]. This hypothesis was tested using an ITC displacement assay, whereby the binding of H11 to Stg-bound FLArc-7A was measured (Fig 8C, Table 3). FLrArc-7A bound the Stg peptide with a *K*_*d*_ of 34.9 *μ*M in an enthalpy-driven manner, similarly to the isolated hArc N-lobe [34]. Thus, the peptide binding affinity of the N-lobe is not altered in the full-length protein, confirming that the poly-Ala mutation in FLrArc-7A did not affect binding. Upon titration of H11 into Stg-bound FLrArc-7A, the measured binding enthalpy corresponded to that of H11 with the binding enthalpy of Stg subtracted (Table 3). However, the ΔG (and *K*_*d*_) of H11 binding to Stg-bound FLrArc-7A was unchanged, due to more favourable binding entropy (Table 3). Hence, what was measured was the binding of H11, accompanied by the dissociation of Stg, demonstrating that H11-induced displacement of the Stg peptide. As it has a K_d_ of ~1nM towards Arc, NbArc-H11 is a tool that could be used to inhibit protein-protein interactions involving Arc *in vivo*, to study the role of these interactions in Arc-mediated regulation of synaptic plasticity.

**Table 3:** ITC displacement assay to follow H11 and Stg binding to FLrArc-7A. The thermodynamic parameters of binding were obtained from the titration curves in Fig 8C. Error margins indicate errors of data fitting from single experiments.

Titration	Number of sites	*K*_*d*_	ΔH (kJ mol^-1^)	-TΔS (kJ mol^-1^)	ΔG (kJ mol^-1^)
H11	0.892±0.003	1.12±0.25 nM	-107.57±0.51	56.28	-51.29
Stg	1.090±0.011	34.90±0.2 *μ*M	-38.50±0.64	15.54	-22.96
H11 into FLrArc-7A+Stg	0.834±0.002	0.87±0.35 nm	-75.23±0.89	24.92	-50.31

### Exploring the dynamic nature of the Arc-CTD central helix hinge region

**Fig 9:**
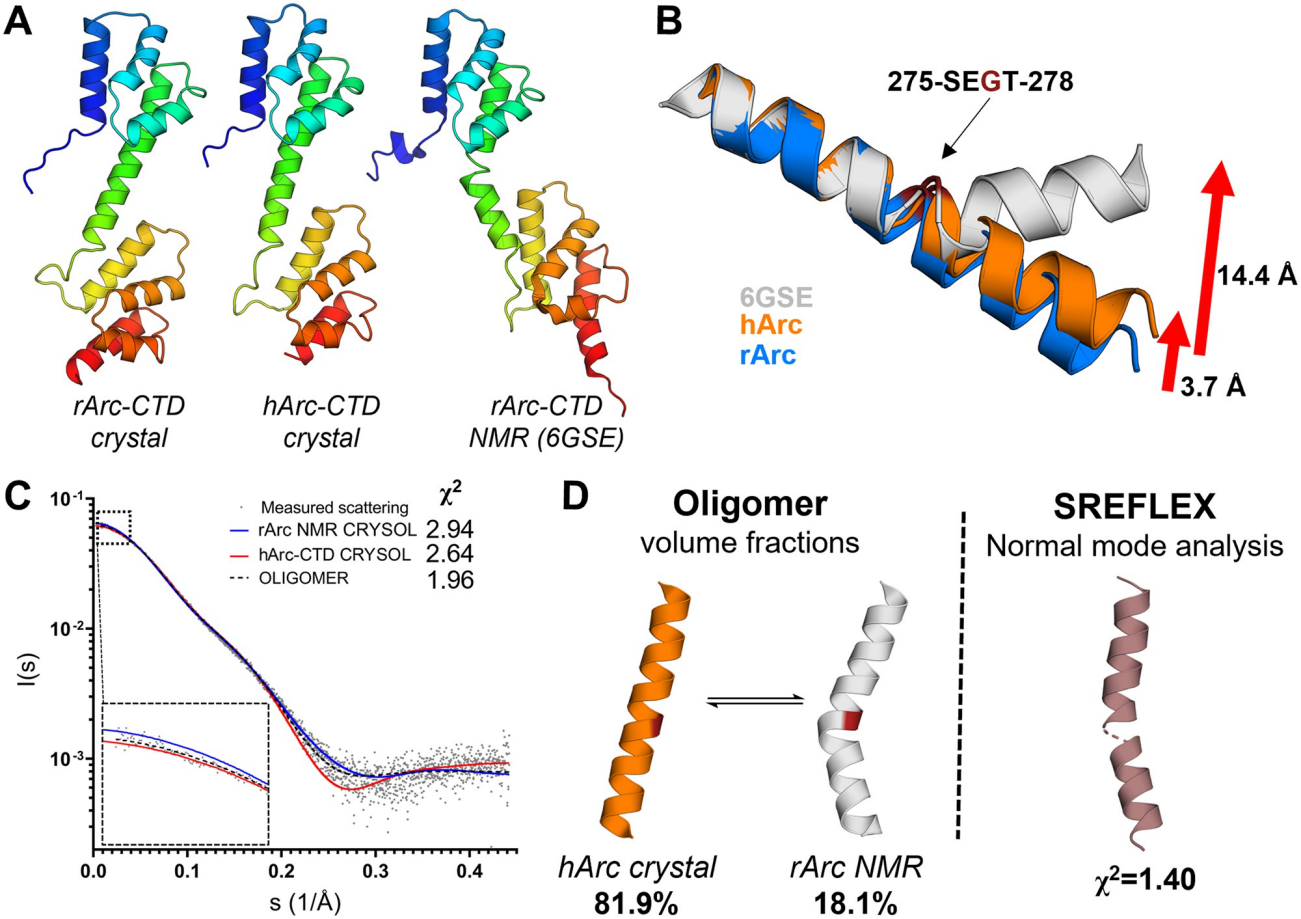
The Arc-CTD is dynamic in structure. **A** Comparison of the Arc-CTD crystal structures with the NMR model of rArc-CTD (6GSE, [31]) reveals a hinge in the central helix. **B** Superimposition of the N-terminal end of the helix highlights a hinge at Gly277. **C** Analysis of the solution structure of the Arc-CTD using SAXS. Shown are the experimental scattering curve of hArc-CTD, theoretical curves for the crystal structure and the NMR model (6GSE, [31]), and the fit from volume fraction. **D** Central helix conformation and results of volume fraction analysis (left). Normal mode analysis (right) of the hArc-CTD crystal structure against the SAXS data gave an average solution conformer of the central helix. The N- and C-lobes are not shown in panels C and D for clarity.

The crystal structures of the human and rat Arc CTD showed almost identical folds, except in the conformation of the central helix. In the rArc structure, the helix is straight (Fig 7A), whereas in hArc, it is kinked (panel A in S5 Fig), resulting in dislocation of the C-lobe in relation to the N-lobe. Upon comparison with the NMR model of the CTD from rArc [31], this conformational variation becomes even more apparent (Fig 9A). Breaking of the helix at Gly277 results in a conformational shift, corresponding to a 3.7 Å translation of the C-terminal end of the helix in the hArc crystal and 14.4 Å in the NMR model (Fig 9B). The dynamics of the central helix might have a role in the function of Arc, and dynamic movements of the CTD could differ between monomeric/dimeric Arc, with potential roles in synaptic plasticity, and the capsid form, which facilitates intercellular mRNA transport [11]. The corresponding helix in both dArc1 and dArc2 breaks, as the two lobes of CTD pack against each other in the capsid protomers [40]. To further explore this hypothesis, SAXS and MD simulations were carried out.

To estimate the conformational flexibility of the CTD in solution, SAXS was used (Fig 9C and 9D). Fitting of theoretical scattering curves from the hArc-CTD crystal structure and the NMR model of the rArc-CTD against SAXS data from hArc-CTD [35] showed that neither seemed fully representative of the solution structure. Simultaneous fitting of both conformers gave fractions of around 80% and 20% for the crystal and NMR conformations, respectively. Hence, the mean solution conformer was close to the crystal structure, but not identical. Normal mode analysis of the hArc-CTD crystal structure against the SAXS data gave an average conformer that showed a slightly kinked helix. Thus, considerable conformational flexibility is present in the dynamic CTD in solution.

To obtain a more detailed insight into conformational plasticity of the CTD, the hArc-CTD crystal structure, stripped of bound Nbs, was subjected to a 640-ns atomistic MD simulation (S7 Fig). In the simulation, the breaking of the central helix at Gly277 facilitates extension of the central hinge, and after rotation, the two lobes pack onto each other, resulting in collapse after only 50 ns. This collapsed conformation seemed more stable than the extended conformer, as little conformational change was observed for the remainder of the simulation. Therefore, the simulation suggested the CTD to be dynamic in structure and implied that hArc-CTD can fold into conformers similar to the capsid protomers of dArc1 and dArc2.

Thr278 of the Arc-CTD undergoes phosphorylation by TNIK kinase in Neuro2A cells, and a phosphomimicking mutation reduces the ability of recombinant mouse Arc to form capsids [78]. Furthermore, the G277D mutation was suggested to reduce the ability of Arc to oligomerise [42]. As both mutations are located in the dynamic hinge region of the CTD central helix, MD simulations were run for G277D and T278E, to examine if altered dynamics would be observed (S7 Fig). In G277D, the helix did break but full compaction was restricted by the negative charge on Asp277. In the T278E phosphomimic, Glu278 formed a salt-bridge with the Arg281 to restrict extension of the hinge region, inhibiting the collapse of the domain. As these mutations reduce Arc to oligomerisation, the simulations suggest a role for the CTD structural dynamics in Arc capsid assembly.

### Crystal structure of a collapsed Arc-CTD

**Fig 10:**
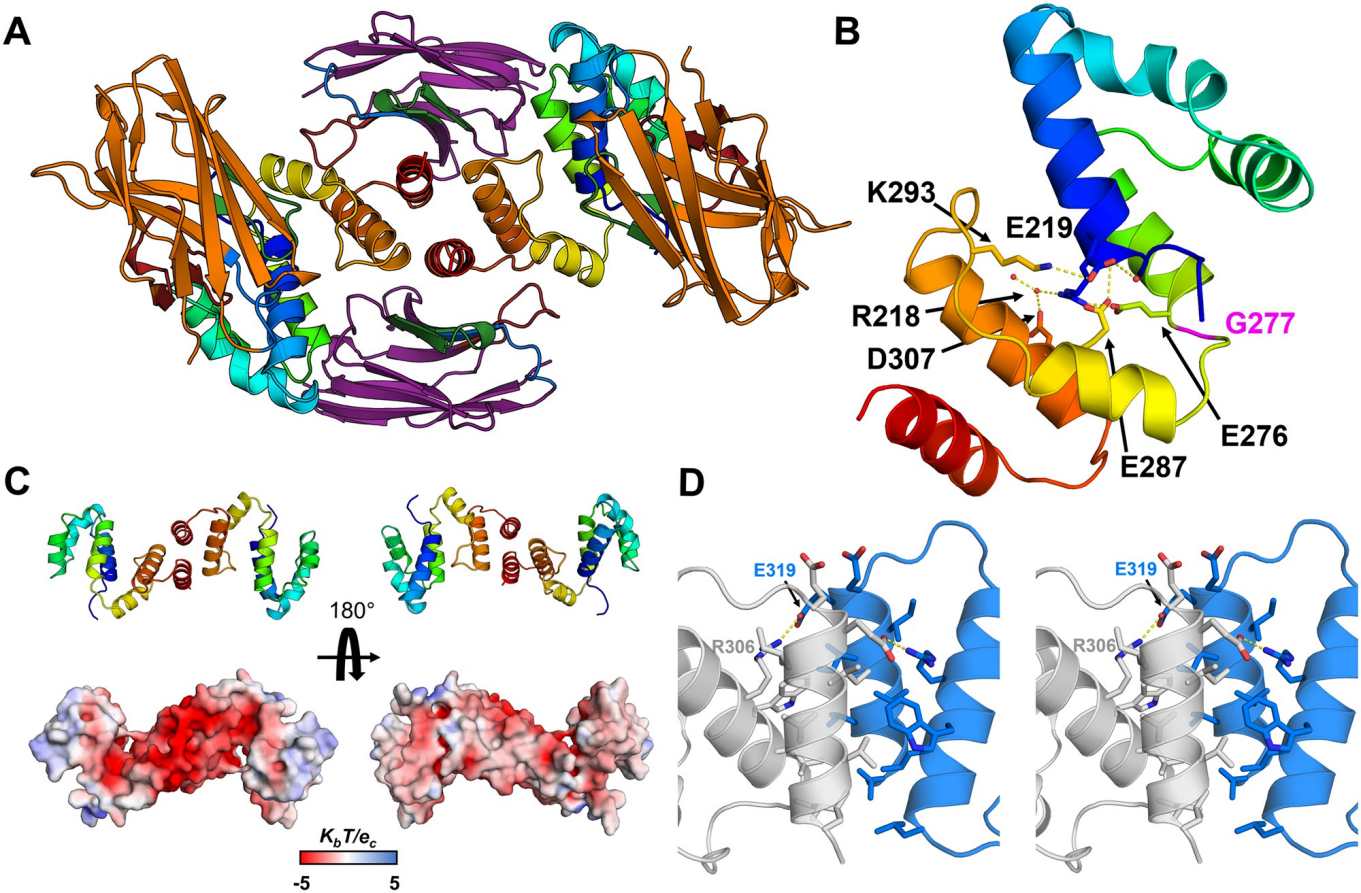
Structure of the collapsed CTD of hArc. **A** The overall structure of the dimeric hArc-CTD+H11+C11 complex, viewed down the dimer symmetry axis. The two CTDs are shown in rainbow, H11 in orange, and C11 in purple. CDR1, 2, and 3 are coloured blue, green, and red, respectively. **B** A monomer, highlighting the collapse of the CTD and lack of the last two helices of the C-lobe. Shown are the sidechains with interactions on the interface. Gly277, at the central helix hinge, is shown in magenta. **C** Dimerisation results in rearrangement of charges, resulting in one highly acidic surface. **D** The CTD dimer interface (stereo view). The residues that form the interface are shown, including the inter-monomer salt bridge between Glu319 and Arg306.

Considering the dynamic nature of the Arc-CTD, crystallisation of the protein in different conformers was attempted. Optimisation of previously neglected crystallisation conditions for hArc-CTD in complex with NbArc-H11 and -C11 and matrix microseeding produced crystals for structure determination, allowing for structure determination at 1.94-Å resolution (Fig 10 and S8 Fig).

The complex crystallised in a different unit cell, when compared to the other structures of the rat and human Arc CTD, and the structure of the complex was different (Fig 10A). In this conformer, breaking of the central helix at Gly277 leads to a complete collapse of the CTD and packing of the C-lobe against the N-lobe (Fig 10B). The interface between the lobes remained partially hydrated and was mainly characterised by indirect hydrogen bonding or salt bridges. Moreover, the CTD appeared dimeric. Using PISA, the calculated free energy gain upon dimerisation (ΔG^int^) of -54.4 kJ mol^-1^ indicated a stable dimeric assembly. The putative dimer interface (Fig 10D), which buries 600 Å^2^ of surface area from each monomer, formed between the C-lobes and involves 10 residues of each subunit. Residues 212–330 of the CTD could be built into the electron density; the last two helices were missing. Therefore, the dimer interface formed between incomplete C-lobes, fusing the exposed hydrophobic cores of the two protomers. Accordingly, the dimer interface is dominated by non-polar interactions. What led to the absence of the C-terminal end of the CTD, and dimerisation, is unresolved. Either the missing C-terminal portion was lost due to degradation, or it was not observed due to flexibility. Large empty spaces were observed in the crystal lattice in proximity of the C-termini (panel C in S8 Fig). The observed dimerisation led to a rearrangement of the electrostatic potential of the CTD, resulting in one side of the dimer being highly acidic (Fig 10C). This might facilitate interactions with cationic components, such as the Arc NTD. The unique CTD conformer observed in the crystal resembles that observed in MD simulations and could be representative of the hArc capsid protomer, as the conformation closely resembles a variety of capsid protomers, including dArc1 and dArc2 [40].

With the ability of Nbs to select for unique protein conformers, it was surprising that both NbArc-H11 and -C11 bound to both the extended and collapsed conformer (Fig 10A). In particular, a portion of the C11 epitope, present on the last two helices of the C-lobe, was absent in the structure, and the surface area C11 buries from the C-lobe was reduced to 658 Å^2^. This was compensated by interactions of the FR of C11 with the N-lobe of the CTD, not observed in the other crystal structures, with an additional interacting surface of 271 Å^2^. Whether these additional interactions are representative of binding interactions in solution is uncertain. In addition, the binding of H11 did not restrict the conformational shift. Upon the collapse of the CTD, the second loop of the C-lobe (the ⍺5-⍺6 loop of the CTD) comes into proximity of the CDR2 loop of H11, showing modest interactions but no steric hindrance. Otherwise, the overall binding mode of H11 was nearly identical to what was observed in the other crystal structures.

### Solution structures of hArc-CTD-Nb complexes

Considering the lack of strict conformational selection by NbArc-H11 and -C11, the solution structures of all Nbs in complex with Arc CTD were investigated using SEC-SAXS (S9 and S10 Figs, Table 4). The aim was to determine whether conformational selection occurred and allow for coarse epitope mapping. In all cases, the Nbs bound monomeric hArc-CTD in 1:1 stoichiometry, suggesting that the dimeric hArc-CTD+H11+C11 crystal structure was either an artefact of crystallisation, or that it only occurred following the loss of the C-terminal end of the CTD.

**Table 4:** Size and shape parameters for CTD-Nb complexes and rArc-NTD, based on SAXS.

Protein	hArc-CTD	+H11	+C11	+E5	+D4	+B5	+B12	MBP-2rNT
R_g_ (Å)	23.0	25.3	24.6	24.5	25.5	24.4	23.7	38.79
Porod volume (nm^3^)	29.22	43.02	40.37	40.44	42.51	42.07	38.93	155.0
P(r) R_g_ (Å)	23.4	25.7	25.0	24.9	26.0	24.7	24.2	39.91
D_max_ (Å)	83.0	86.5	85.2	85.1	94.0	83.5	82.0	125.15
Quality est. GNOM	0.71	0.87	0.83	0.80	0.72	0.87	0.85	0.90
Q_p_ Mw (kDA)	21.4	32.1	29.9	30.1	30.6	31.5	28.8	109.9
Monomer Mass (kDA)	21.7	35.8	34.9	34.5	34.6	34.9	34.4	56.0
Oligomeric state/stoichiometry	Monomeric	1:1	1:1	1:1	1:1	1:1	1:1	Dimer

In complex with the Nbs, the CTD seemed to compact to some extent, as the Kratky plots (panel C in S9 Fig) suggested that the complexes were more compact than the free CTD. R_g_ only slightly increased and D_max_ remained almost unchanged despite the increased Porod volume and molecular weight, which both increase by ~50% upon Nb binding (Table 4). This was observed most clearly for the B12 complex. As the Nbs approximate the hArc-CTD in mass, one would have expected a greater size increase in the complexes. Compactness was further highlighted in the calculated distance distribution of the complexes (panel D in S9 Fig), where the split profile in the unbound protein, characteristic of a bilobar structure, is lost into a single wider peak, accompanied by little change in D_max_. The CTD compaction upon Nb binding is apparent in the *ab initio* models of the complexes (S10 Fig). The models of the complexes were similar in length to the free CTD, while the linker in the apo domain was not as apparent. Although epitopes could not be mapped with SAXS due to the symmetric nature of the CTD, it seemed that all Nbs associated with either the N- or the C-lobe. Altogether, SAXS showed that Nb binding, to either of the two lobes, drives compaction of the CTD *via* conformational selection. Compaction of the CTD in full-length Arc might be facilitated by interdomain interactions with the NTD.

### The coiled-coil NTD mediates dimerisation of FLrArc-7A, indicating a role in higher-order assembly

**Fig 11:**
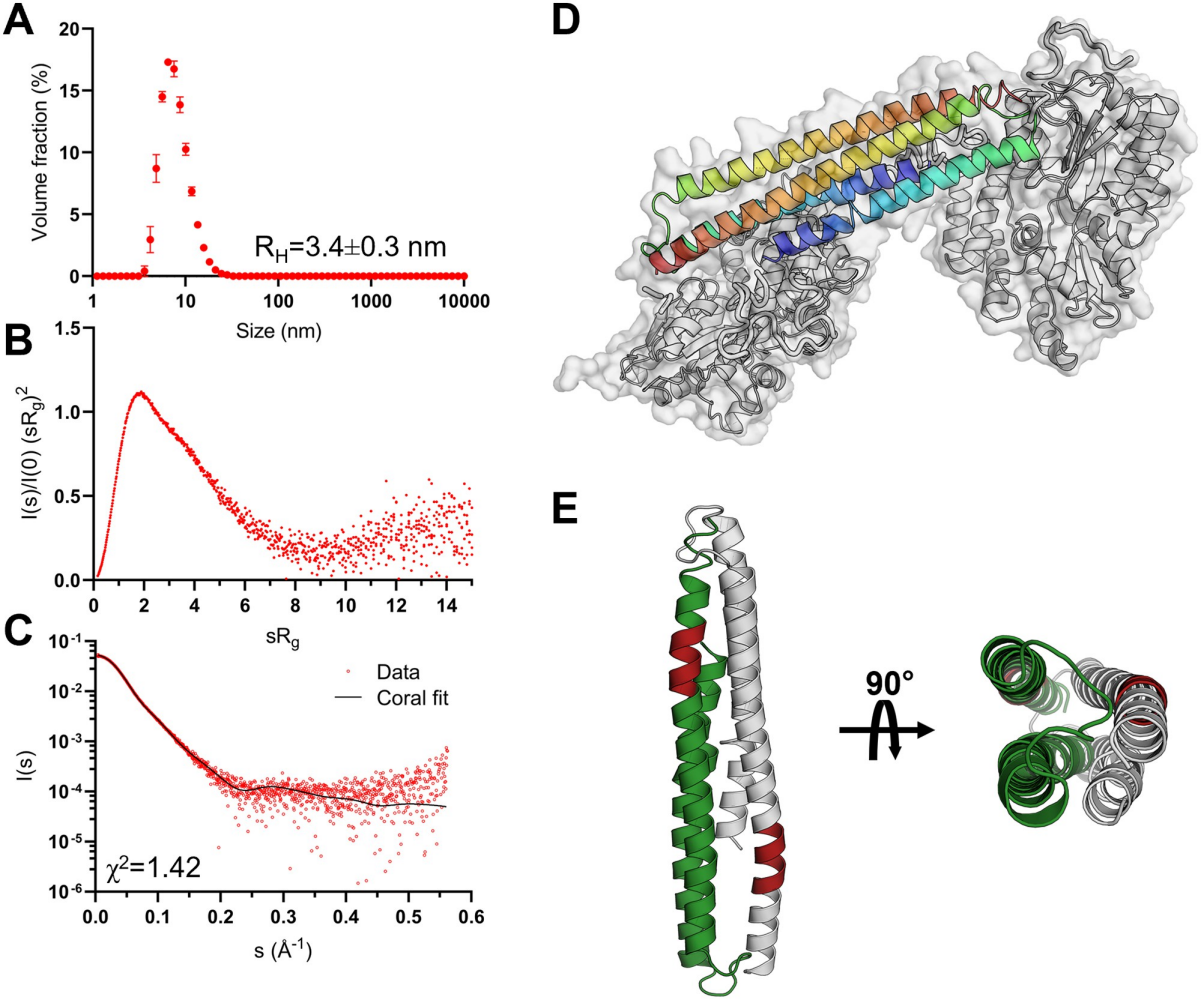
Biophysical characterisation and modelling of the soluble MBP-fused NTD construct. **A** DLS size distribution of the fusion protein at 1 mg/mL, showing a single monodisperse species with an apparent hydrodynamic radius (R_H_) of 3.4 nm. **B** Dimensionless Kratky plot derived from SAXS data, shown in C, highlighting the monodisperse and rigid nature of the protein. **C** MBP-2rNT SAXS scattering curve, superimposed with the CRYSOL fit obtained in modelling of the NTD dimer. **D** *In silico* model of the dimeric MBP-2rNT construct made in CRYSOL from SAXS data, using a homology model of the NTD [35] and a crystal structure of MBP [79]. **E** The arrangement of the NTD in the model, showing a 4-helix bundle of antiparallel coiled coils. The oligomerisation motif (mutated in this construct) is shown in red.

To assess the role of the individual domains of FLrArc-7A in dimerisation and higher-order oligomerisation, solution structures and oligomeric states were assessed using SAXS. The Arc-NTD is poorly soluble, and the isolated domain resisted recombinant expression and purification. Therefore, rArc-NTD with the oligomerisation-inhibiting poly-Ala mutation was produced as a soluble MBP fusion (MBP-2rNT) for SAXS experiments (Table 4, Fig 11, S11 and S15 Figs). MBP-2rNT was dimeric and rigid, in contrast to the monomeric hArc-CTD (Table 4, Fig 11A and 11B). This indicated that dimerisation of full-length FLrArc-7A is likely mediated by dimerisation of the NTD. The *ab initio* model of MBP-2rNT (S11 Fig) revealed a rod-shaped fold with lobes on opposing ends, which represent the N-terminal MBP fusions at opposing N-termini. The density at the centre of the envelope therefore accounts for the Arc NTD, predicted as a four-helix bundle formed by head-to-tail coiled coils. This model of MBP-2rNT was further explored with *in silico* modelling in CORAL, using the homology model of the NTD [35] and a crystal structure of MBP (Fig 11C and 11D). In this conformation, the two oligomerisation motifs that were mutated in our construct, ^113^MHVWREV^119^, are located at opposite ends of the four-helix bundle (Fig 11E). Together with the dynamic nature of the CTD, the model provides insights into the mechanism of Arc oligomerisation.

To confirm that the Arc NTD was folded in the MBP fusion, we carried out additional characterisation. Using L-Arg as a solubiliser, we could, indeed, purify soluble NTD from the fusion protein after TEV cleavage (S16 Fig). However, the low yield shifted our focus to using the MBP fusion, which allowed us to detect 2-fold symmetry mediated by the Arc NTD and localise the Arc N terminus *via* the folded MBP (Fig 11D). The purification of the different soluble constructs, including MBP-2rNT, showed that out of them all, MBP-2rNT had the highest content of ⍺-helix (S15 Fig), arguing against misfolding or unfolding of the Arc NTD within the construct. Furthermore, subtracting the CTD CD data from the FLrArc7A spectrum showed that the NTD is helically folded in the full-length construct (panel D in S15 Fig). The quantitative formation of the NTD-mediated homodimer of the fusion protein, proven by SAXS data, also shows the Arc NTD is folded in the construct.

## Discussion

We produced and characterised six high-affinity anti-Arc Nbs for Arc binding and validated their use as crystallographic chaperones and effectors of Arc interactions. In parallel, we are validating the Nbs for a variety of methods and functional studies [80]. Two of the Nbs, NbArc-H11 and C11 were used to obtain crystal structures of the rat and human Arc-CTD. Our results provide an insight into the structural dynamics of the CTD in higher-order oligomerisation and capsid formation. The new information obtained on Arc structure and its implications on Arc function are discussed below.

### Peptide binding displacement and its effects on Arc structure

**Fig 12:**
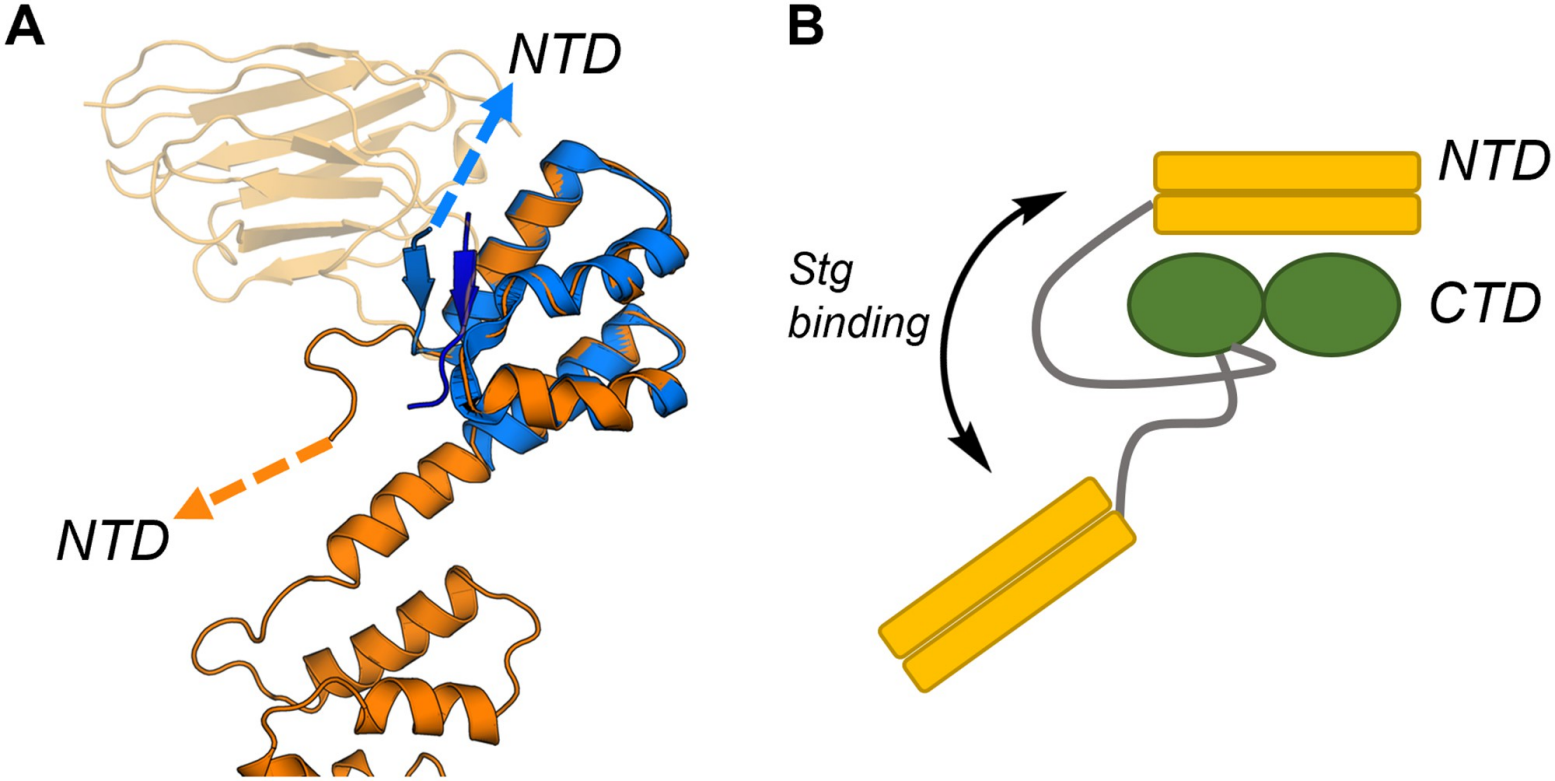
The potential effect of Stg binding on the structure of full-length Arc. **A** Alignment of the extended conformer of the hArc-CTD (orange), crystallised in complex with NbArc-H11 and -C11, and the isolated hArc-CTD N-lobe crystallised in complex with Stg (blue, 6TNO, [34]). NbArc-H11 is shown in transparent orange and the bound Stg in dark blue. The conformational change in the linker region is noted, by arrows pointing towards the NTD. **B** The hypothesised effect of Stg binding on the structure of linker region and the relative orientation of the two domains of Arc. The linker region is shown in grey.

The Arc-CTD binds ligand peptides in a hydrophobic groove of the N-lobe [26,34]. The CDR3 of NbArc-H11 contains the conserved motif of the Arc ligand motif and bound in the same binding site with nanomolar affinity. We further demonstrated that H11 could efficiently displace the Stg peptide bound to the FLrArc-7A CTD. In the structure of the hArc-CTD N-lobe in complex with the Stg peptide, the portion of the NTD-CTD linker preceding the CTD N-terminus folds against the peptide bound in the hydrophobic groove of the N-lobe [34]. In the structure of the hArc-CTD, obtained here *via* co-crystallisation with NbArc-H11 and -C11, the same segment extends away from the N-lobe, similarly to the solution structure of Arc-CTD [31] (Fig 12A). These findings show that ligand peptides can alter the structure of the NTD-CTD linker region, possibly resulting in large changes in the relative orientation of the domains of Arc (Fig 12B). This could represent a previously unrecognised mechanism of how Stg and other Arc interaction partners might regulate the interdomain interactions of full-length Arc and higher-order oligomerisation.

### The CTD conformational flexibility and its relevance

The hArc-CTD crystallised in the extended conformation at a high concentration (37 mg/mL) with Nbs H11 and C11, suggesting a solubilising effect by Nb binding. However, at a lower concentration (10 mg/mL), the complex crystallised in a collapsed conformer. What led to the crystallisation of this unique conformer of the hArc-CTD, is uncertain. Possibly, the low ionic strength of the crystallisation condition had an effect; capsid formation by Arc is dependent on ionic strength [20]. A more likely explanation lies in the absence of the C-terminal end of the CTD. Loss of the last two helices of the hArc-CTD resulted in dimerisation, in which the exposed hydrophobic cores of the remaining C-lobes formed the dimer interface. Interestingly, this dimerisation mode resulted in one highly acidic face, while both C-termini localised on the opposite side. In the capsids formed by dArc1, the 48-residue C-terminal tail is located inside of the capsid. Two tails of the hexameric capsomer subsequently form a two-stranded zinc finger to facilitate RNA binding, while the remaining four remain unstructured [40]. Similarly, the collapsed conformer of the hArc-CTD could represent the structure of the CTD in hArc capsids, whereby the acidic side binds the basic NTD on the outer capsid layer, while the C-terminal tail locates to the inside. Moreover, phosphorylation sites have been identified in the in unstructured C-terminal portion of Arc; phosphorylation of Thr368 and Thr380 regulates the degradation of Arc [81,82]. Whether these post-translational modifications facilitate the unfolding of the C-terminal end of the CTD and the subsequent dimerisation, and possibly the higher-order oligomerisation of Arc, remains a subject of further study.

### Insights into Arc capsid formation

Using NbArc-H11 and -C11 as crystallisation chaperones, rArc CTD was crystallised. The same Nbs allowed for the crystallisation of the hArc-CTD in both an extended and a collapsed conformation. This dynamic nature of the CTD was further investigated using SAXS and MD simulations, which both provided evidence for a hinge region. In MD simulations of the G277D and T278E variants, the conformational plasticity was lost and the CTD remained extended. As these mutations, and especially the T278E phosphomimic, were reported to reduce the ability of full-length mArc to form capsids [42,83], our data suggest a role for the structural plasticity of the CTD, and the collapsed form observed in the crystal structure, in capsid formation. Notably, AlphaFold2 [84] predicts both the extended and collapsed forms of hArc-CTD, lending further support to both conformations being functionally significant (S12 Fig).

**Fig 13:**
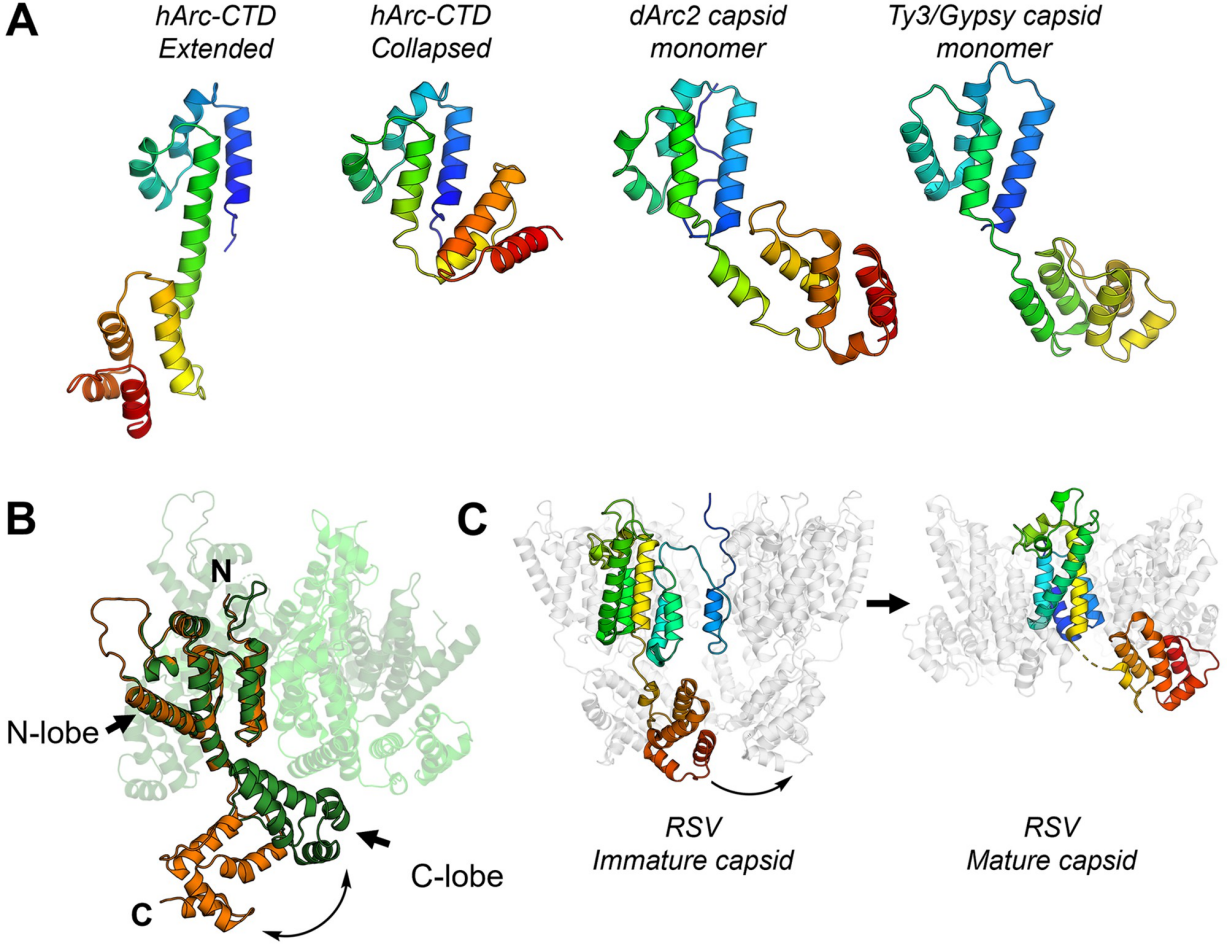
CTD hinge region flexibility is conserved in other LTR retrotransposons and retroviral CA domains. **A** Comparison of the extended and collapsed crystal structures of the hArc-CTD, obtained in this study, with the dArc2 (6TAQ, [40]) and Ty3/Gypsy (6R24, [85]) capsid monomer reveal varying conformations of LTR retrotransposon capsid proteins. All are aligned on the N-lobe and shown in rainbow. See S13 Fig for an overall alignment of hArc-CTD and dArc2. **B** Comparison of the monomeric HIV CA, crystallised in complex with a Fab fragment (orange, 1E6J, [88]), and the CA in the capsid pentamer (3P05, [86]) reveal structural plasticity of the linker region, similar to that observed in the Arc-CTD. The HIV CA monomer is shown in orange and the capsid pentamer in green. The capsid protomers not aligned with the monomeric CA are shown in various shades of green transparent cartoons. **C** Structure of immature RSV viral particles (5A9E, [89]) and mature RSV capsid pentamers (7NO5, [90]) shows similar structural fluctuations in the process of capsid assembly.

The similarity of the N- and C-lobes of dArc and mArc-CTD with retroviral Gag polyprotein capsid (CA) domains has been demonstrated [26,34,39,41]. In the dArc capsids, and the capsid formed by the homologous Ty3/Gypsy retrotransposon [40,85], the two lobes are connected by a short linker, similar to that observed in the collapsed crystal form of hArc-CTD (Figs 13A and S13). From the capsid structures, it seems that breaking of the CTD linker region is needed for capsid assembly. Similarly to Arc and Ty3/Gypsy, the capsid domain (CA) of the HIV Gag polyprotein contains two lobes connected by a short linker. Flexibility of this linker is vital for HIV capsid assembly [86,87]. Moreover, comparison of the monomeric HIV CA domain crystallised in complex with a Fab fragment [88] and the pentameric capsid protomer [86] reveals a conformational change, similar to that observed here for Arc-CTD, between the monomeric and capsid forms (Fig 13B). The same applies to Rous Sarcoma Virus (RSV), where an extended conformer is found in immature viral particles, and a hinge region facilitates formation of mature RSV capsids [89,90] (Fig 13C). These observations suggest that the structural plasticity of the Arc-CTD might represent a conserved mechanism for capsid assembly of retroviruses and long terminal repeat (LTR) retrotransposons. In this respect, it should be remembered that our experiments on Arc-CTD conformation were carried out in the absence of the NTD, which could affect the interdomain dynamics within the Arc-CTD in solution and in the capsid form.

The capsids of dArc1 and dArc2 consist of penta- and hexameric capsomers, the formation of which depends on homo-oligomerisation of the N-lobe, while assembly of the mature capsid is facilitated by dimerisation of the C-lobe [40]. This is in line with the strong tendency of the individual dArc lobes to homo-oligomerise [41]. In contrast, the CTD of mArc is monomeric in solution, as also observed here for hArc [35]. A determining factor of mArc capsid assembly is the oligomerisation motif in the second coil of the coiled-coil NTD [18]. However, the NTD does not independently assemble into capsids in the absence of the CTD, indicating a role for both domains in capsid assembly [20]. Previous studies have suggested domain swapping of the NTD and CTD to be a determining factor in oligomerisation and capsid assembly [35]. However, the structural plasticity of hArc-CTD and its similarity to other retrotransposons and capsid proteins (Fig 13) suggest that it might form penta- and hexameric capsomers in an icosahedral capsid, similarly to dArc. Moreover, SAXS data of the MBP-fused, mutated NTD construct showed that rArc-NTD is a homodimer. In a model of the NTD (Fig 11), the oligomerisation motifs are situated at opposing ends of the dimer.

Based on current data, a model for hArc capsid formation can be speculated upon, in which the dimerisation and further oligomerisation of the NTD facilitate capsomer formation and link adjacent capsomers. The capsomeric CTD may be similar to the collapsed hArc-CTD crystal structure, and additional dimerisation of the CTD C-lobe cannot be excluded. The NTD binds to and induces curvature in anionic membranes [35,36], and it could be situated on the outside of the capsid to facilitate intercellular transport. While other models of capsid assembly exist, this hypothesis explains its dependency on the NTD oligomerisation motif and accounts for both NTD dimerisation and CTD structural plasticity. Dimerisation *via* the NTD could be achieved through domain swapping, in agreement with earlier hypotheses [35]. This approximates the inter-capsomer domain swapping observed in Simian virus 40 and polyomavirus capsids [91,92]. In such a model, the 84-residue linker between the NTD and CTD would have to traverse from the centre of the capsomer towards the dimerising NTDs. The average end-to-end distance of an 84-residue disordered chain is around 8.3 nm [93], and the length of the linker could, theoretically, extend up to ~30 nm. Its length should, therefore, not be a limiting factor. The validation of such a model remains a subject of further study.

## Anti-Arc Nbs as tools for functional studies

Nbs are powerful tools for probing protein structure, function, and dynamics. The monomeric and soluble nature of Nbs allows for, in many cases, their correct folding and function in the reducing environment of the eukaryotic cytosol. Moreover, as they only consist of a single domain, the manipulation of their sequence and addition of various fusion proteins becomes possible. As an example, transgenic expression of a green fluorescent protein (GFP) -fused Nb, a so-called chromobody, can allow for real-time visualisation of subcellular localisation of the antigen in live cells [94], and co-expression of Nbs fused to subunits of the E3-ubiquitin ligase complex rapidly facilitates ubiquitinoylation and degradation of the target protein [95,96]. In addition, the conformationally selective nature of Nb binding has been utilised to stabilise transient, physiologically relevant conformers and to probe conformational dynamics *in vitro* and *in vivo* [97,98]. Arc has several putative interaction partners in the PSD, including Stg, GluN2A, GKAP [26,31,34]. The physiological relevance of these interactions, however, remains poorly understood. The H11 Nb characterised here is a tool for studying these interactions, as it efficiently displaces bound peptide without affecting Arc structural integrity or capsid formation by purified Arc.

In parallel with the present work, we have generated recombinant epitope-tagged Nbs for immunoblotting and affinity-purification of endogenous Arc from brain tissue samples, including Arc expressed after induction of synaptic plasticity (LTP) in live rats [80]. Epitope mapping, based on expression of Arc segments in cell lines, shows that H11 and E5 selectively bind the N-lobe, while B5, B12, C11, and D4 bind the C-lobe [80]. When expressed as genetically encoded intrabodies, H11 and E5 bound to the N-lobe and allowed immunoprecipitation of full-length Arc. In addition, Nbs fused to the fluorescent protein mScarlet expressed uniformly in cell compartments without aggregation, indicating their suitability as chromobodies [80]. We foresee a variety of applications of Arc Nbs as modular tools for studying Arc function, subcellular localisation, and structural dynamics *in vivo*.

## Concluding remarks

We have presented a series of recombinant high-affinity nanobodies directed against the N- and C-lobes of the mammalian Arc CTD. As shown here, these nanobodies are functional tools for structural biology and biochemical approaches, and they can be further developed towards applications in *e*.*g*. imaging and functional modulation of Arc [80]. From the structural studies enabled by the nanobodies, we have obtained crystal structures of the mArc CTD in two different conformations, highlighting a hinge region in the linker helix between the CTD lobe domains as possibly important for Arc conformational changes and virus-like capsid formation. Future work using these nanobody tools will enable novel, exciting research avenues in structural and functional biology of mammalian Arc.

## Supporting information

S1 Fig Flexibility of the CDR loops of NbArc-E5. (PDF)

S2 Fig NbArc ITC titrations into FLrArc-7A, raw and integrated thermograms. (PDF)

S3 Fig Competition assay of FLrArc-7A with selected nanobodies using analytical SEC. (PDF)

S4 Fig Crystal contacts. (PDF)

S5 Fig Crystal structure of hArc-CTD in complex with NbArc-H11 and -C11. (PDF)

S6 Fig Raw ITC thermograms. (PDF)

S7 Fig MD simulations of wild-type (WT) CTD with and without bound Stg and mutants G277D and T278E. (PDF)

S8 Fig Crystal structure of the broken hArc-CTD conformer in complex with NbArc-H11 and -C11. (PDF)

S9 Fig SEC-SAXS of hArc-CTD Nb complexes. (PDF)

S10 Fig *Ab initio* models of the hArc-CTD in apo state and in complex with the anti-Arc Nbs derived from the SAXS data. (PDF)

S11 Fig SEC-SAXS analysis of MBP-2rNT, and the other two Arc constructs used in this study. (PDF)

S12 Fig AlphaFold2 reproduces both the extended and collapsed conformation of hArc-CTD. (PDF)

S13 Fig Superposition of collapsed conformations. (PDF)

S14 Fig Unedited gel figures. (PDF)

S15 Fig Purifications and CD. (PDF)

S16 Fig Small-scale purification of Arc-NTD after cleavage of MBP tag. (PDF)

S1 Table X-ray diffraction data collection and refinement statistics. (DOCX)

S1 Protocol Arc expression and purification. (DOCX)

S2 Protocol Small-angle X-ray scattering data collection. (DOCX)

S3 Protocol Crystallisation and structure solution of individual proteins and complexes. (DOCX)
