# Supplementary material for: High-affinity anti-Arc nanobodies provide tools for structural and functional studies
Source: PLoS One. 2022 Jun 7;17(6):e0269281. doi: 10.1371/journal.pone.0269281 (PMC9173642; doi:10.1371/journal.pone.0269281)
Supplement: S1 Fig — (PDF) [file pone.0269281.s001.pdf]

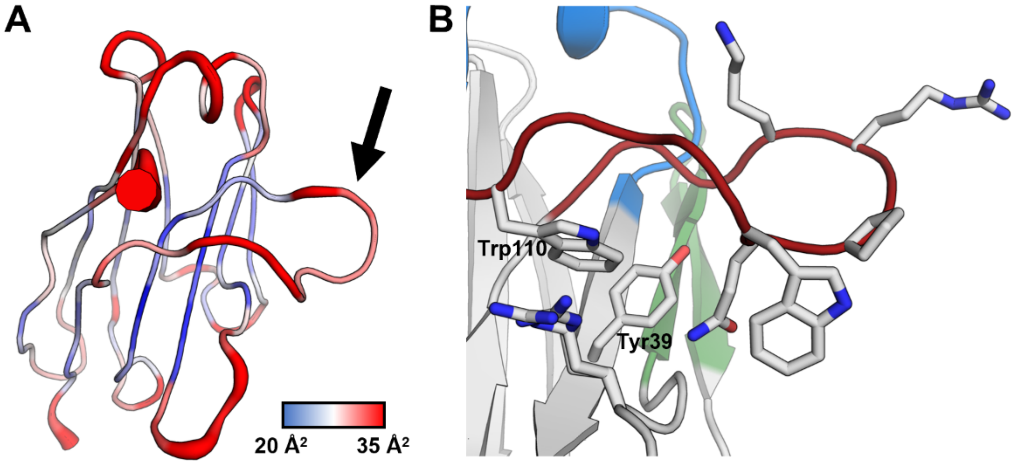

S1 Figure. Flexibility of the CDR loops of NbArc-E5. A The crystallographic B-factors of the NbArc-E5 crystal structure demonstrate the rigidity of the CDR3 loop (highlighted by arrow). **B** Rigidity of the CDR3 loop is likely accounted for by packing of its large hydrophobic side chains onto the exterior of the β-barrel fold. This could also account for the increased solubility of NbArc-E5 when compared to the other Nbs.
