# Supplementary material for: High-affinity anti-Arc nanobodies provide tools for structural and functional studies
Source: PLoS One. 2022 Jun 7;17(6):e0269281. doi: 10.1371/journal.pone.0269281 (PMC9173642; doi:10.1371/journal.pone.0269281)
Supplement: S2 Fig — (PDF) [file pone.0269281.s002.pdf]

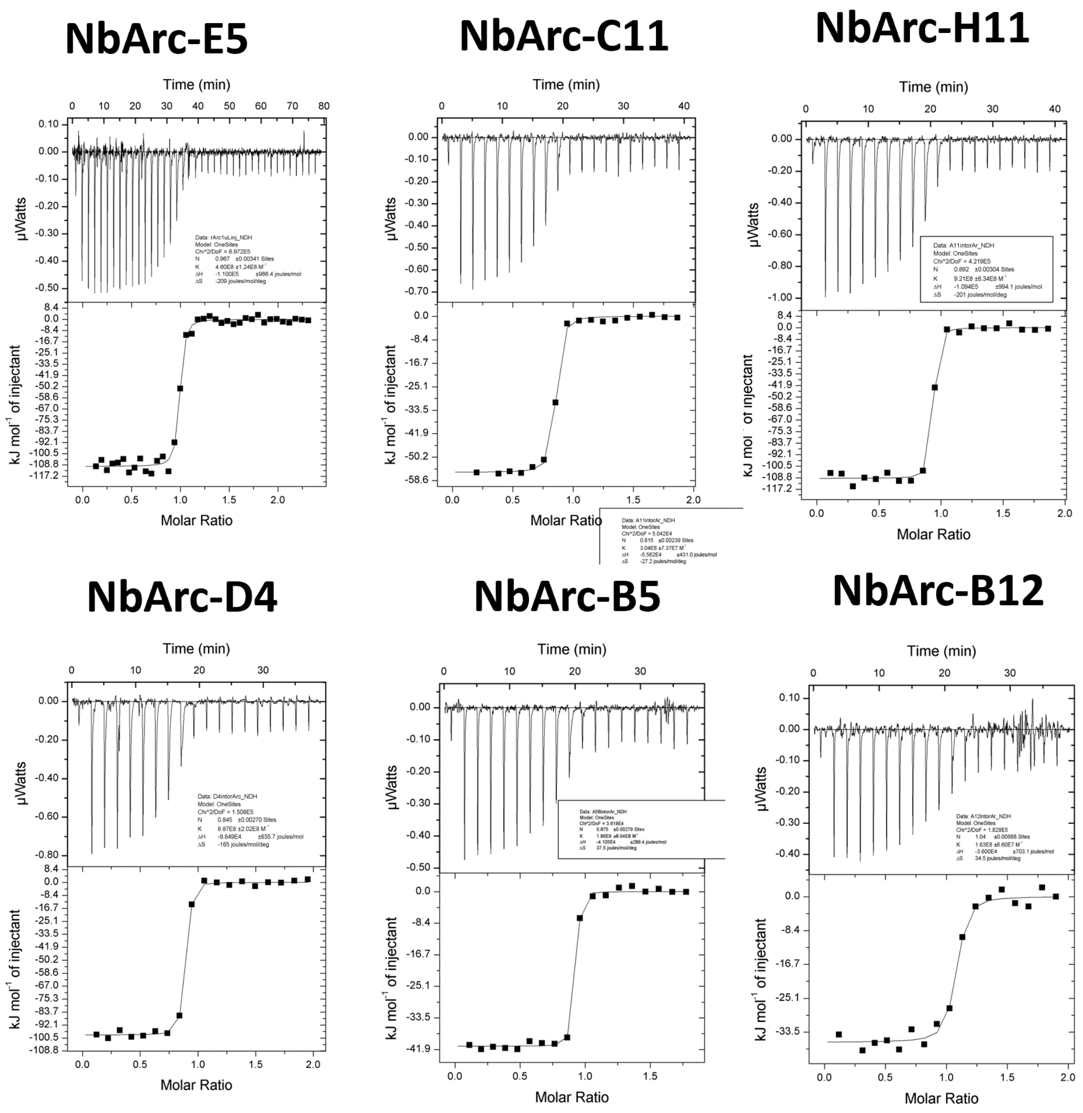


**S2 Figure. NbArc ITC titrations into FLrArc-7A, raw and integrated thermograms.**
