# Supplementary material for: High-affinity anti-Arc nanobodies provide tools for structural and functional studies
Source: PLoS One. 2022 Jun 7;17(6):e0269281. doi: 10.1371/journal.pone.0269281 (PMC9173642; doi:10.1371/journal.pone.0269281)
Supplement: S3 Fig — (PDF) [file pone.0269281.s003.pdf]

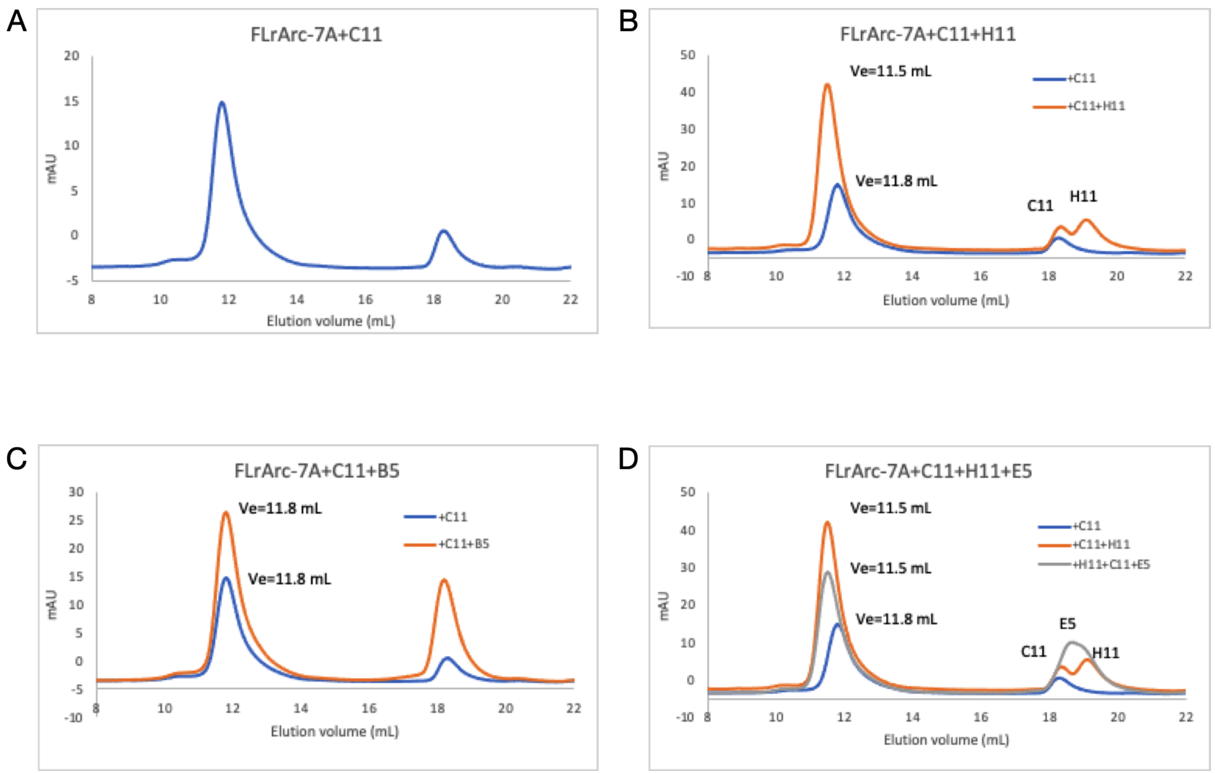

S3 Figure. Competition assay of FLrArc-7A with selected nanobodies using analytical SEC. A The complex with C11 elutes at ~12 ml and the excess Nb at ~18 ml. **B** Position of the complex peak reveals that both C11 and H11 bind at the same time. **C** C11 and B5 compete for binding, as no shift of the complex peak is observed. **D** H~~C~~11 and E5 compete for binding.
