# Supplementary material for: High-affinity anti-Arc nanobodies provide tools for structural and functional studies
Source: PLoS One. 2022 Jun 7;17(6):e0269281. doi: 10.1371/journal.pone.0269281 (PMC9173642; doi:10.1371/journal.pone.0269281)
Supplement: S4 Fig — (PDF) [file pone.0269281.s004.pdf]

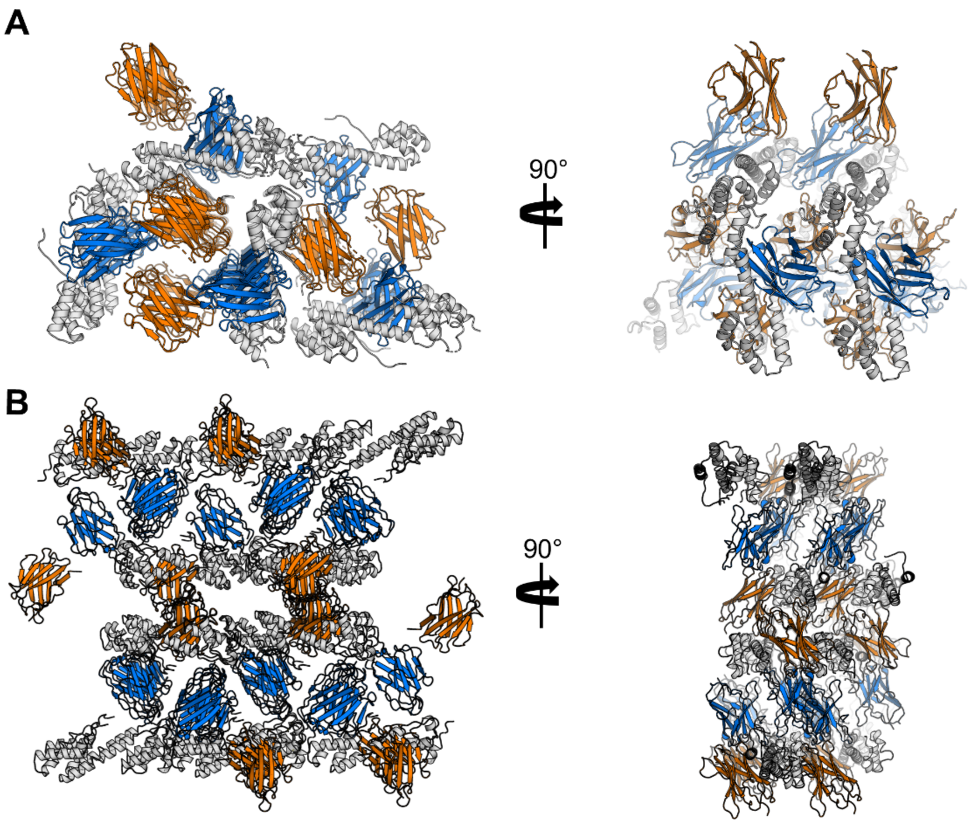


**S4 Figure. Crystal contacts.** In both the rArc-CTD (**A**) and the hArc-CTD extended crystal structure (**B**), contacts were formed almost exclusively by the bound Nbs. The CTD is coloured grey, H11 orange and C11 blue.
