# Supplementary material for: High-affinity anti-Arc nanobodies provide tools for structural and functional studies
Source: PLoS One. 2022 Jun 7;17(6):e0269281. doi: 10.1371/journal.pone.0269281 (PMC9173642; doi:10.1371/journal.pone.0269281)
Supplement: S5 Fig — (PDF) [file pone.0269281.s005.pdf]

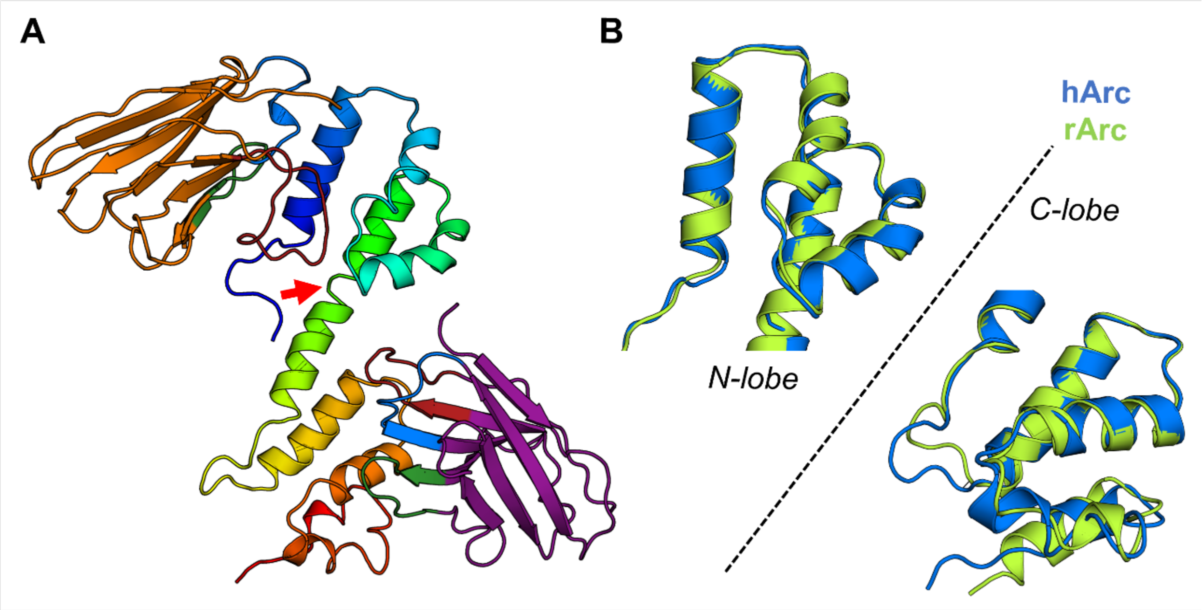

S5 Figure. Crystal structure of hArc-CTD in complex with NbArc-H11 and -C11. A The structure of the ternary complex. The breaking of the central helix of the CTD is highlighted with a red arrow. The hArc-CTD is coloured blue to red from N- to C-terminus. H11 is shown in orange and C11 in purple. CDR 1, 2, and 3 are coloured blue, green, and red, respectively. **B** Superimposition of the N- and C-lobes of rArc and hArc CTD from the crystal structures.
