# Supplementary material for: High-affinity anti-Arc nanobodies provide tools for structural and functional studies
Source: PLoS One. 2022 Jun 7;17(6):e0269281. doi: 10.1371/journal.pone.0269281 (PMC9173642; doi:10.1371/journal.pone.0269281)
Supplement: S6 Fig — (PDF) [file pone.0269281.s006.pdf]

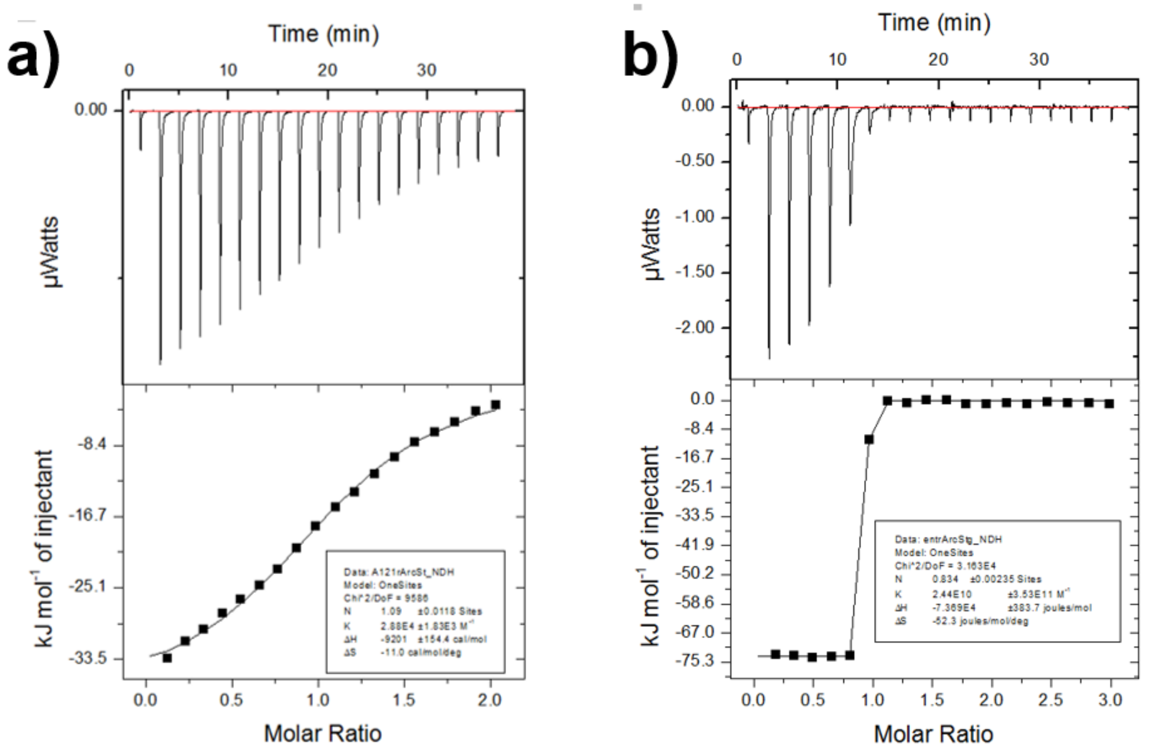

S6 Figure. Raw ITC thermograms. A Titration of the Stg peptide into FLrArc-7A. **B** titration of NbArc-H11 into Stg-bound FLrArc-7A.
