# Supplementary material for: High-affinity anti-Arc nanobodies provide tools for structural and functional studies
Source: PLoS One. 2022 Jun 7;17(6):e0269281. doi: 10.1371/journal.pone.0269281 (PMC9173642; doi:10.1371/journal.pone.0269281)
Supplement: S7 Fig — (PDF) [file pone.0269281.s007.pdf]

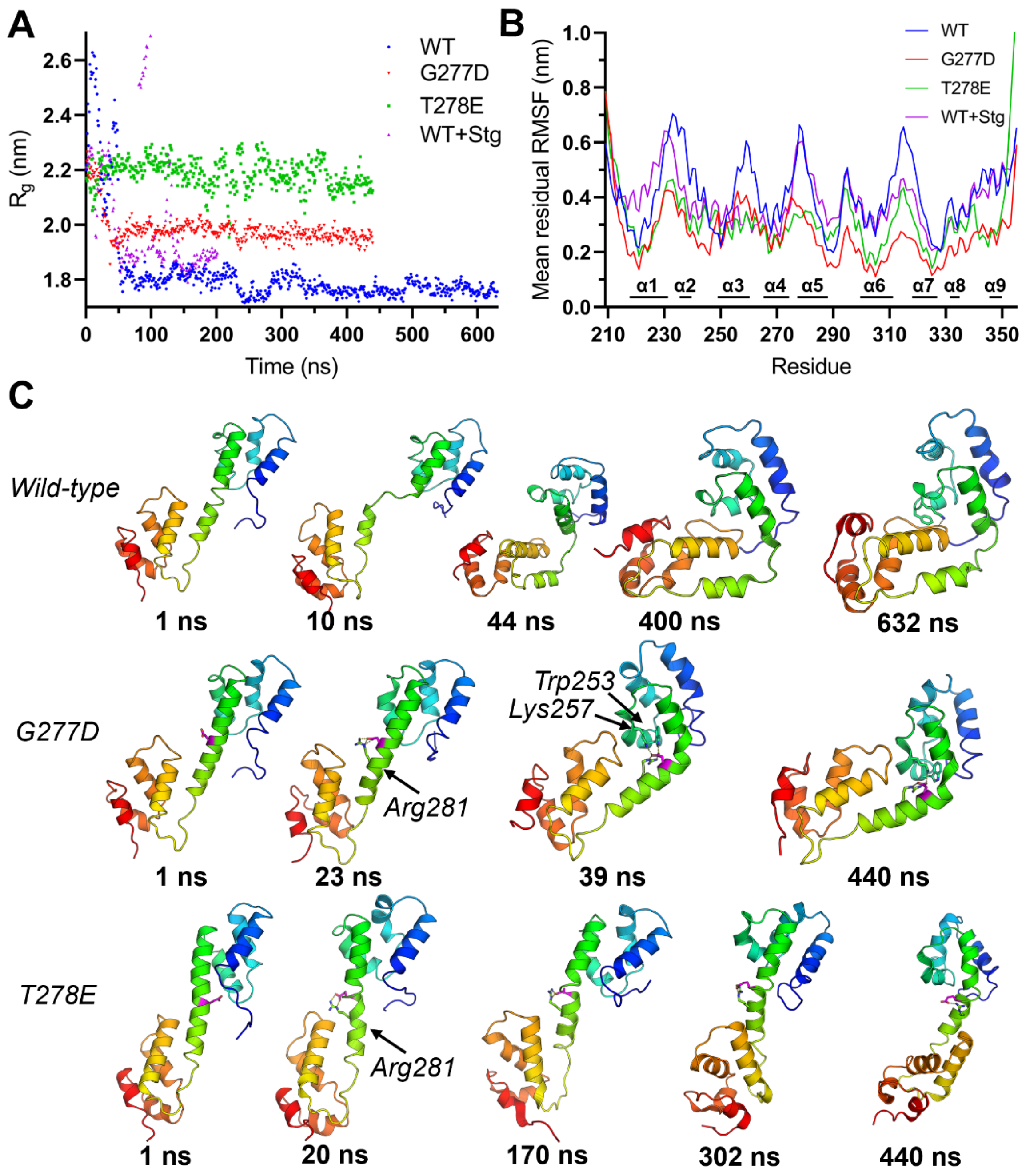

S7 Figure. MD simulations of wild-type (WT) CTD with and without bound Stg and mutants G277D and T278E. A R_g over the trajectories demonstrates compaction of the wild-type CTD and lack of it in the G277D and T278E mutants. **B** Mean residual RMSF demonstrates the reduced flexibility of the mutants. **C** Trajectory snapshots at selected time points. The *in silico* mutated residues in G277D and T278E are highlighted in magenta.
