# Supplementary material for: High-affinity anti-Arc nanobodies provide tools for structural and functional studies
Source: PLoS One. 2022 Jun 7;17(6):e0269281. doi: 10.1371/journal.pone.0269281 (PMC9173642; doi:10.1371/journal.pone.0269281)
Supplement: S8 Fig — (PDF) [file pone.0269281.s008.pdf]

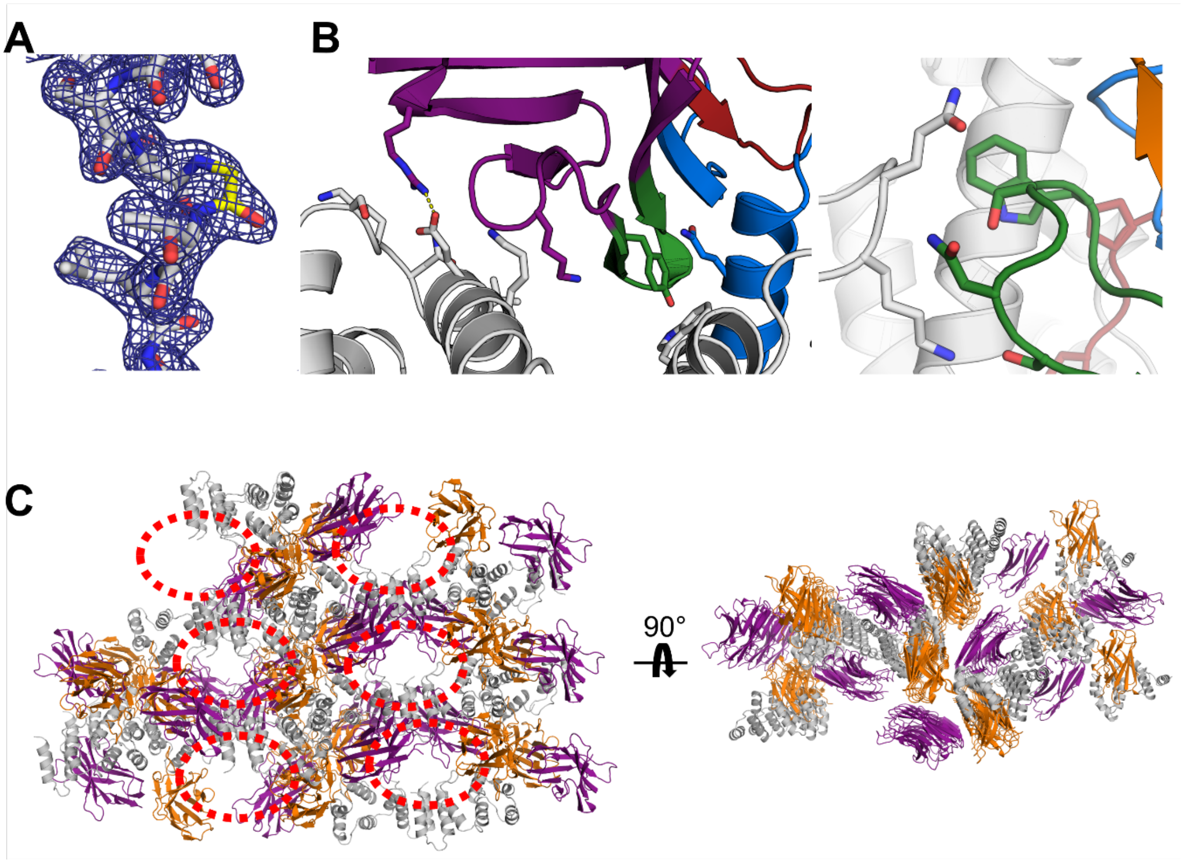

S8 Figure. Crystal structure of the broken hArc-CTD conformer in complex with NbArc-H11 and -C11. **A** Electron density map ($2F_O$-F_C at 1.5σ in blue) of the hinge region where Gly277 is highlighted in yellow, demonstrating the lack of ambiguity in building the broken hinge region. **B** The additional Nb interactions observed in the crystal. FR region of C11 (left) binds the N-lobe and H11 (right) shows modest interactions with the collapsed C-lobe. **C** Packing in the crystal, where crystal contacts are exclusively formed by bound Nbs. Empty volumes in the lattice which might contain the unstructured C-terminal portion of the CTD are highlighted with red broken circles.
