# Supplementary material for: High-affinity anti-Arc nanobodies provide tools for structural and functional studies
Source: PLoS One. 2022 Jun 7;17(6):e0269281. doi: 10.1371/journal.pone.0269281 (PMC9173642; doi:10.1371/journal.pone.0269281)
Supplement: S9 Fig — (PDF) [file pone.0269281.s009.pdf]

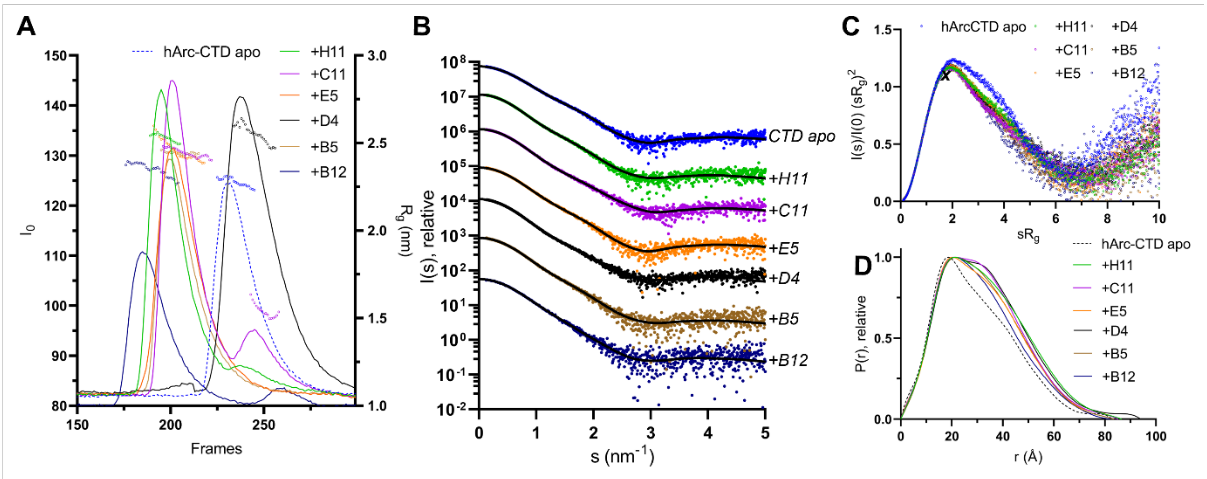

S9 Figure. SEC-SAXS of hArc-CTD Nb complexes. A SEC-SAXS elution profiles. The observed scattering intensity is shown as lines (left Y-axis) and the calculated R_g is shown for the frames used for data processing. R_g of the Nb excess peaks is shown for C11 and H11+C11 complex run, although they were not used for processing. **B** Scattering curves obtained from the main SEC peaks. Curves are offset for clarity, and data fits from GNOM shown as solid lines. **C** Dimensionless Kratky plot. The maximum for an ideal rigid spherical particle is marked with ***X*** ($\sqrt{3}$, 1.104). **D** Distance distribution profiles. The free hArc-CTD (apo) profile is shown as black dashes.
