# Supplementary material for: High-affinity anti-Arc nanobodies provide tools for structural and functional studies
Source: PLoS One. 2022 Jun 7;17(6):e0269281. doi: 10.1371/journal.pone.0269281 (PMC9173642; doi:10.1371/journal.pone.0269281)
Supplement: S11 Fig — (PDF) [file pone.0269281.s011.pdf]

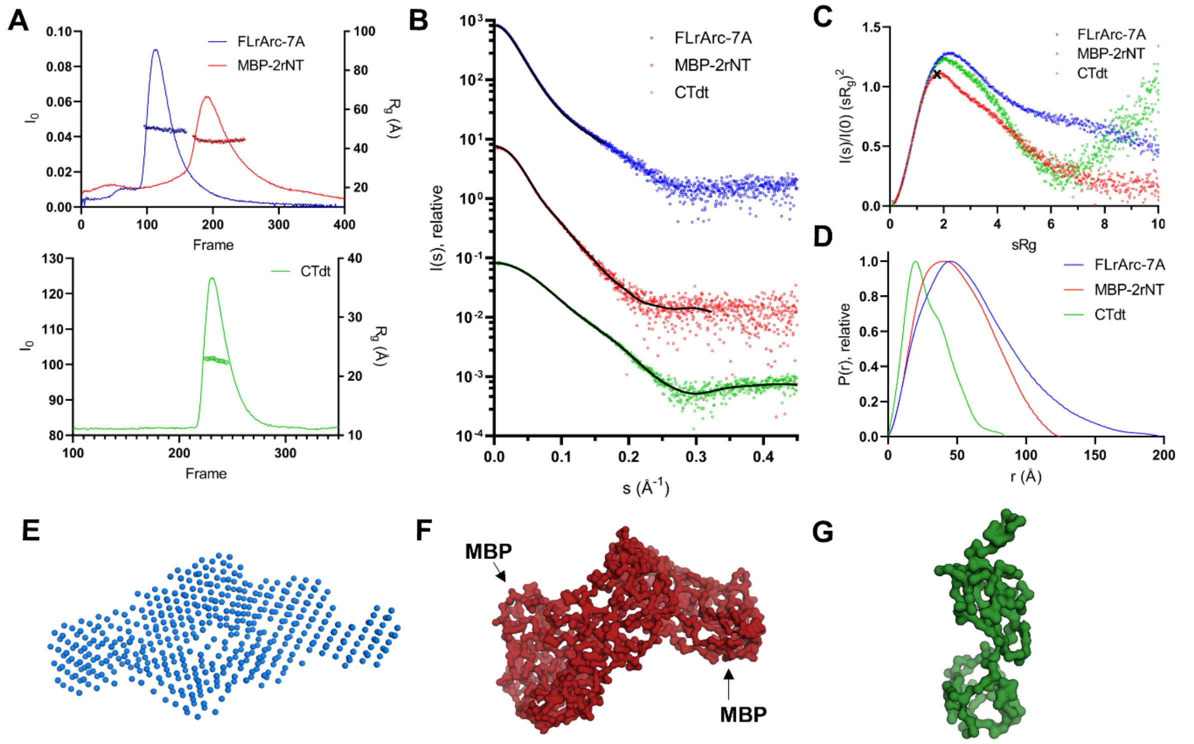

S11 Figure. SEC-SAXS analysis of MBP-2rNT, and the other two Arc constructs used in this study. CTdt refers to the hArc-CTD construct used. **A** SEC elution profiles for the three constructs. Extrapolated I0 of each frame (lines) are plotted on the left Y-axis and open rings indicate the calculated R_g of the main peak data frames used for data processing and modelling. **B** Scattering profiles of each sample. The data fits from GNOM are shown as black lines and the curves are displaced by two logarithmic units, for clarity. **C** Scattering data shown on a dimensionless Kratky plot. The X indicates the expected maximum for a fully rigid spherical particle (√3, 1.104). The rigidity of the MBP-2rNT construct was apparent. **D** Distance distribution profiles. FLrArc-7A showed a characteristic wide profile, indicating elongation, hArc-CTD showed a two-peak profile, typical of bilobar structures and the bell-shaped distribution of MBP-2rNT indicated a more compact fold. **E** *Ab initio* model of the dimeric rArc mutant, produced with no forced symmetry in DAMMIN (χ^2=0.9992). *Ab initio* models of the MBP-2rNT dimer (**F**) and hArc-CTD (**G**), produced using GASBOR without forced symmetry (χ^2=1.790 for MBP-2rNT and χ^2=1.261 for hArc-CTD).
