# Supplementary material for: High-affinity anti-Arc nanobodies provide tools for structural and functional studies
Source: PLoS One. 2022 Jun 7;17(6):e0269281. doi: 10.1371/journal.pone.0269281 (PMC9173642; doi:10.1371/journal.pone.0269281)
Supplement: S12 Fig — (PDF) [file pone.0269281.s012.pdf]

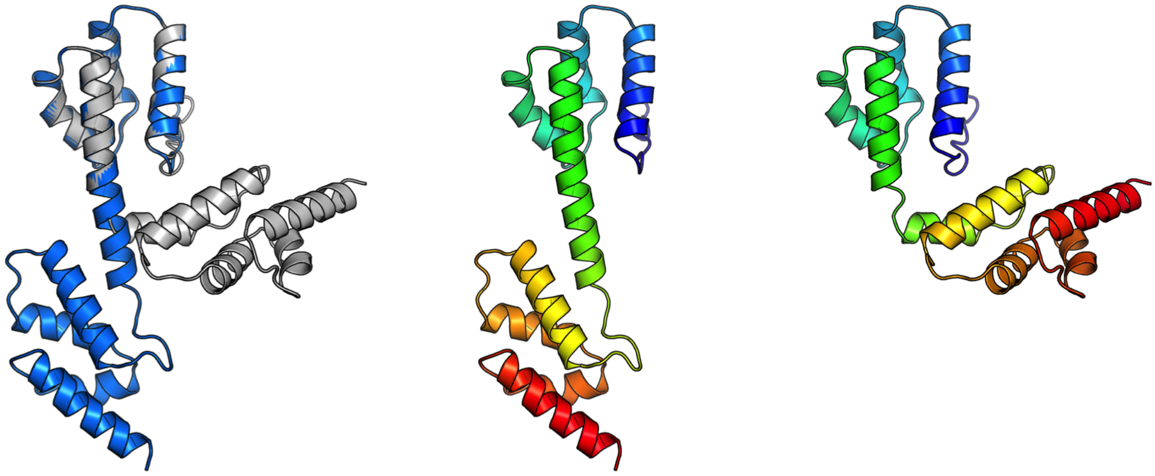

S12 Figure. AlphaFold2 reproduces both the extended and collapsed conformation of hArc-CTD. Left: superposition of models ranked #1 (blue) and #5 (gray). Models #2-4 were essentially identical to #1. Middle: the extended conformation. Right: the collapsed conformation. The two conformations predicted correspond remarkably well to the observed structures in the ternary complexes.
