# Supplementary material for: High-affinity anti-Arc nanobodies provide tools for structural and functional studies
Source: PLoS One. 2022 Jun 7;17(6):e0269281. doi: 10.1371/journal.pone.0269281 (PMC9173642; doi:10.1371/journal.pone.0269281)
Supplement: S13 Fig — (PDF) [file pone.0269281.s013.pdf]

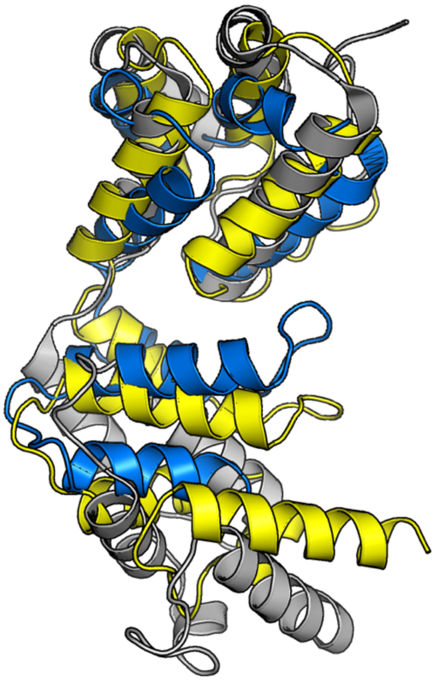

S13 Figure. Superposition of collapsed conformations. The collapsed hArc CTD crystal structure (blue), dArc2 monomer from a capsid (gray), and AlphaFold2 prediction of the collapsed form (yellow).
