# Supplementary material for: High-affinity anti-Arc nanobodies provide tools for structural and functional studies
Source: PLoS One. 2022 Jun 7;17(6):e0269281. doi: 10.1371/journal.pone.0269281 (PMC9173642; doi:10.1371/journal.pone.0269281)
Supplement: S14 Fig — (PDF) [file pone.0269281.s014.pdf]

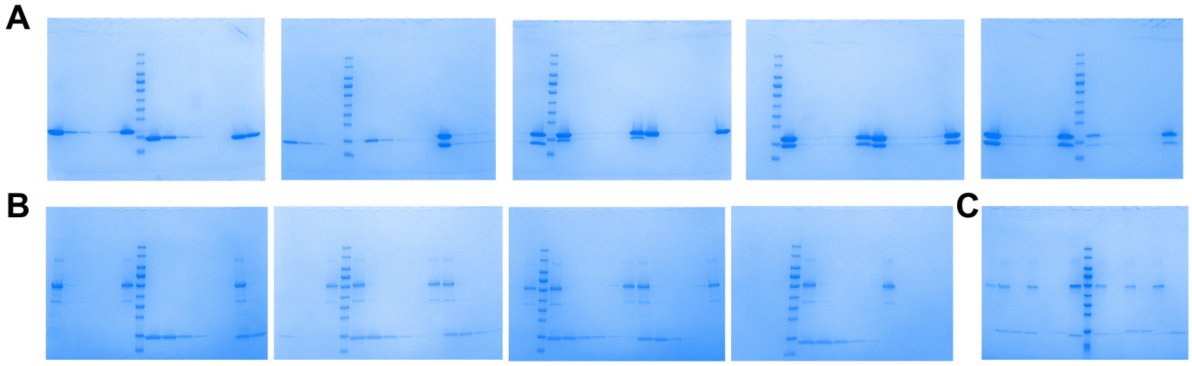

S14 Figure. Unedited gel figures. Unedited SDS-PAGE gel images of (**A**) hArc-CTD Nb pulldowns, (**B**) MBP-2rNT Nb pulldowns and (**C**) hArc capsid Nb co-elutions.
