# Supplementary material for: High-affinity anti-Arc nanobodies provide tools for structural and functional studies
Source: PLoS One. 2022 Jun 7;17(6):e0269281. doi: 10.1371/journal.pone.0269281 (PMC9173642; doi:10.1371/journal.pone.0269281)
Supplement: S15 Fig — (PDF) [file pone.0269281.s015.pdf]

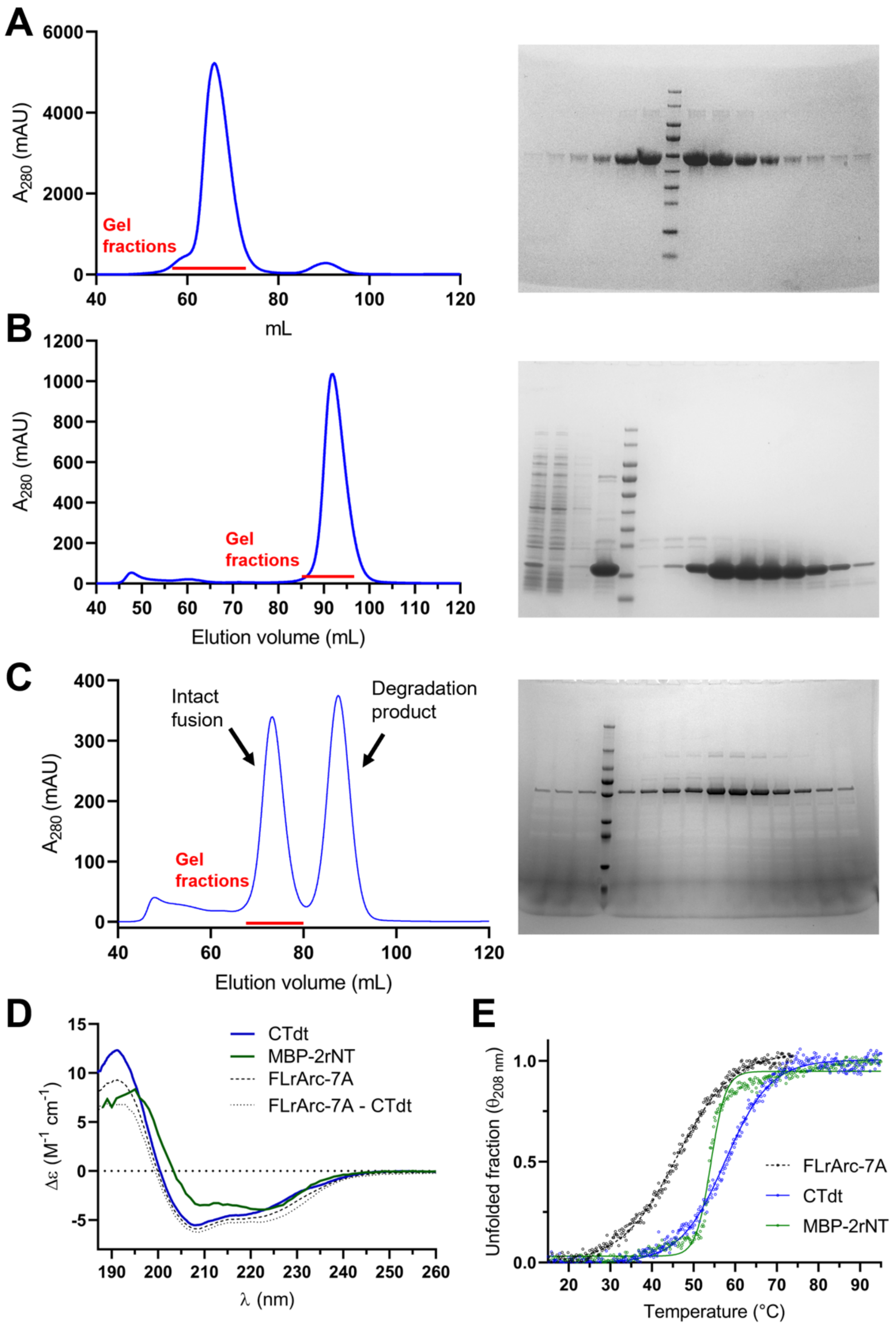

S15 Figure. Purifications and CD. Purification and initial characterisation of the Arc constructs. Purifications of FLrArc-7A, hArc-CTD and the MBP-2rNT fusion are shown in panels **A**, **B** and **C**, respectively. The last SEC purification steps are shown on the left (absorption normalised to 1 cm only for FLrArc-7A), and SDS-PAGE analysis of SEC fractions on the right. **D** CD spectra of the three constructs suggested them all to be mainly α-helical. **E** CD temperature denaturation experiments suggested all three constructs to be well folded, as all gave good denaturation curves. The T_m values derived from the transitions were 46.0°C, 57.6°C and 54.1°C for FLrArc-7A, hArc-CTD and MBP-2rNT, respectively.
