# Supplementary material for: High-affinity anti-Arc nanobodies provide tools for structural and functional studies
Source: PLoS One. 2022 Jun 7;17(6):e0269281. doi: 10.1371/journal.pone.0269281 (PMC9173642; doi:10.1371/journal.pone.0269281)
Supplement: S16 Fig — (PDF) [file pone.0269281.s016.pdf]

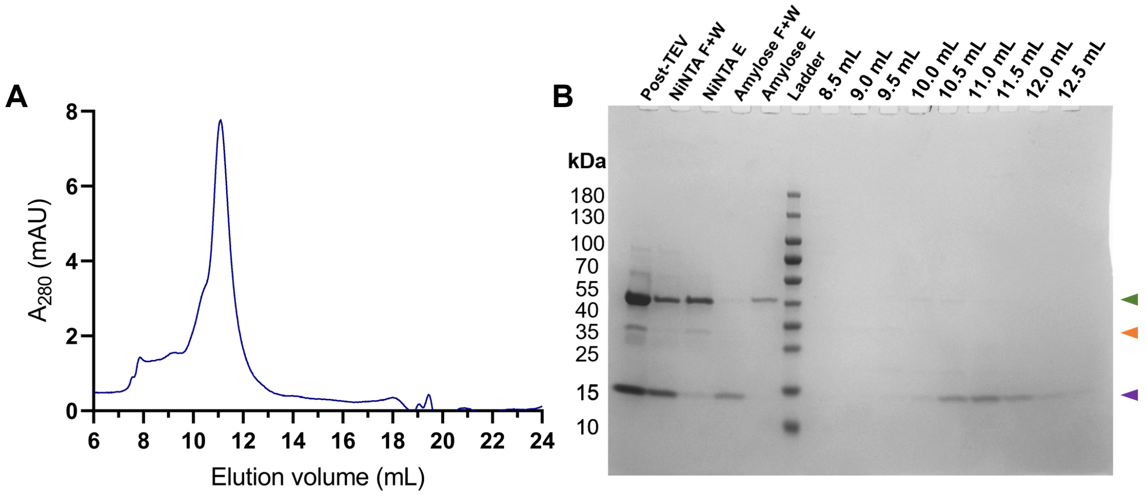

S16 Figure. Small-scale purification of Arc-NTD after cleavage of MBP tag. A. SEC after removal of MBP tag. B. SDS-PAGE of purification and SEC fractions. Indicated are the fusion protein (green arrowhead), MBP (orange), and free Arc-NTD (magenta).
