# Supplementary material for: High-affinity anti-Arc nanobodies provide tools for structural and functional studies
Source: PLoS One. 2022 Jun 7;17(6):e0269281. doi: 10.1371/journal.pone.0269281 (PMC9173642; doi:10.1371/journal.pone.0269281)
Supplement: S1 Table — (DOCX) [file pone.0269281.s017.docx]

**S1 Table**. **X-ray diffraction data collection and refinement statistics.**

Statistics for the highest-resolution shell are indicated in parentheses.

|  | **NbArc-E5** | **rArc+H11+C11** | **hArc+H11+C11, extended** | **hArc+H11+C11,**  **collapsed** |
| --- | --- | --- | --- | --- |
| **Data collection** |  |  |  |  |
| Beamline | I03 | P14 | P11 | P11 |
| Wavelength (Å) | 0.976 | 0.976 | 1.033 | 1.033 |
| Resolution range (Å) | 41.98-1.42 (1.46-1.42) | 38.51-2.70 (2.76-2.70) | 42.99-2.77 (2.84-2.77) | 48.00-1.94 (1.99-1.94) |
| Space group | P4_3_2_1_2 | P2_1_2_1_2_1_ | P2_1_2_1_2_1_ | P2_1_2_1_2 |
| Unit cell parameters | a=b=45.9 Å | a=40.88 Å | a=40.80 Å | a=66.26 Å |
|  | c=103.75 Å | b=92.81 Å | b=61.64 Å | b=139.29 Å |
|  | α=β=γ=90° | c=114.65 Å | c=171.95 Å | c=43.29 Å |
|  |  | α=β=γ=90° | α=β=γ=90° | α=β=γ=90° |
| Redundancy | 9.2 (9.2) | 7.5 (7.8) | 6.6 (6.0) | 11.9 (6.7) |
| Completeness (%) | 99.9 (99.8) | 99.2 (98.2) | 99.6 (99.9) | 86.5 (46.2) |
| Mean I/σ(I) | 9.5 (0.3) | 6.7 (1.0) | 7.5 (0.6) | 13.1 (0.8) |
| CC_1/2_ (%) | 99.9 (33.3) | 99.1 (47.8) | 99.5 (32.4) | 99.9 (35.1) |
| R_meas_ (%) | 10.8 (568.7) | 29.3 (255.2) | 25.1 (340.7) | 13.0 (235.6) |
| Mosaicity (°) | 0.14 | 0.12 | 0.62 | 0.11 |
| Wilson B-factor (Å^2^) | 19.4 | 36.6 | 49.0 | 31.0 |
| **Refinement** |  |  |  |  |
| R_work_ (%) | 19.6 | 25.7 | 25.2 | 19.7 |
| R_free_ (%) | 23.6 | 27.4 | 30.2 | 24.3 |
| RMSD_bonds/angles_ (Å/°) | 0.005/0.72 | 0.002/0.53 | 0.002/0.43 | 0.003/0.61 |
| Ramachandran favoured /outliers (%) | 99.1/0.0 | 92.0/2.1 | 93.4/0.5 | 98.03/0.0 |
| Average B-factor (Å^2^) | 34.7 | 58.8 | 83.6 | 43.8 |
| **PDB code** | 7R20 | 7R24 | 7R23 | 7R1Z |
