# Supplementary material for: High-affinity anti-Arc nanobodies provide tools for structural and functional studies
Source: PLoS One. 2022 Jun 7;17(6):e0269281. doi: 10.1371/journal.pone.0269281 (PMC9173642; doi:10.1371/journal.pone.0269281)
Supplement: S1 Protocol — (DOCX) [file pone.0269281.s018.docx]

**S1 Protocol**

**Arc expression and purification**

FLrArc-7A, full-length rArc containing a poly-Ala mutation (s113-119), was in the pHMGWA vector [1], resulting in an N-terminal His6-maltose binding protein (MBP) fusion [2]. *E. coli* BL21(DE3)-RIPL cells, transformed with the pHMGWA-rArc-FLrArc7A construct, were used to inoculate 10-50 mL LB medium containing 100 μg/mL ampicillin and 34 μg/mL chloramphenicol, followed by overnight culture at +37 °C and 170 rpm. The starter cultures were diluted 100-fold into 1-3 L of the medium and outgrowth carried out at +37 °C and 200 rpm. When the cell density (OD600) reached 0.8-1.0, expression was induced with 1.0 mM isopropyl β-D-1-thiogalactopyranoside (IPTG) and maintained for 4 h at +30 °C and 200 rpm. Cells were harvested via centrifugation at 6,000 g and +4 °C for 25 min, and the pellet was resuspended in 20 mM HEPES pH 7.5, 100 mM NaCl, 1 mM DTT, 0.1 mg/mL hen egg white lysozyme (20 mL per 1 L expression culture). Lysis was carried out via a single freeze-thaw cycle and sonication (seven cycles of 3 x 25 W for 10 s and 0 W for 30 s) and the soluble fraction harvested via centrifugation at 30,000 g and +4 °C for 45 min. The soluble fraction was extruded through 0.45-μm syringe filters and supplemented with 20 mM imidazole pH 7.5 and 1 mM dithiothreitol (DTT) before loading on a Ni^2+^-nitrilotriacetic acid (NiNTA) matrix equilibrated in 20 mM HEPES, 150 mM NaCl, 20 mM imidazole, 1 mM DTT, pH 7.5. The column was washed with 12 column volumes of the same buffer, before eluting the bound protein with the purification buffer containing 300 mM imidazole. To remove the His_6_-MBP tag, tobacco etch virus (TEV) protease was added and the protein dialysed against 1 L of 20 mM HEPES, 150 mM NaCl, 1 mM DTT in 6-8 kDa molecular weight cut-off (MWCO) dialysis tubing at +4 °C overnight. To remove TEV protease, the cleaved fusion tags and undigested fusion proteins, the protein was subjected to negative NiNTA purification following the same procedure as above but omitting imidazole from the washing buffer. The flowthrough and wash fractions were subjected to further negative purification using amylose resin in the same buffer. All affinity steps were performed at +4 °C. The protein was concentrated to 2 mL in a 30 kDa MWCO spin concentrator and applied onto a HiLoad 16/600 Superdex 200 pg size exclusion chromatography (SEC) column (GE healthcare, IL, USA) equilibrated in 20 mM Tris-HCl pH 7.4, 150 mM NaCl. Purity of the SEC fractions was analyzed via SDS-PAGE, before pooling and concentrating to 10-20 mg/mL. The concentrated protein was split into 50 μL aliquots, snap-frozen in liquid N_2_ and stored at -80 °C. Pure proteins were not subjected to more than one freeze-thaw cycle before use in functional assays or crystallisation.

The of human Arc CTD (hArc-CTD) (residues 206-361) was expressed and purified as described [3]. The protein was expressed with an N-terminal His6 tag in E. coli BL21(DE3) cells using the pTH27 vector and purified via affinity chromatography as described for the full-length protein above. As no cleavage of the His6 tag was observed upon incubation with TEV protease, the eluate from the first affinity step was concentrated to 2 mL in a 10 kDa MWCO spin concentrator and further purified on a HiLoad Superdex 75 16/600 pg SEC column equilibrated with 20 mM Tris-HCl pH 7.4, 150 mM NaCl. After SDS-PAGE analysis, the fractions of the main peak were pooled, concentrated to 15-30 mg/mL, aliquoted, snap-frozen in liquid N_2_ and stored at -80°C.

The NTD of FLrArc-7A (2rNT) was expressed as an N-terminal His6-MBP fusion construct in pHMGWA using *E. coli* BL21(DE3)-RIPL cells. A glycerol stock was used to inoculate 30 mL of LB medium containing 100 μg/mL ampicillin and 34 μg/mL chloramphenicol succeeded by overnight incubation at +37 °C and 170 rpm. The starter culture was diluted 100-fold into 3 L of the medium and incubated at +37 °C and 200 rpm. At OD600 = 0.5-0.7, expression was induced with 1.0 mM IPTG and maintained at +18 °C for 16-20 h. Cells were harvested via centrifugation at 6,000 g and 4 °C for 25-40 min, supernatant discarded and the pellet resuspended in 40 mM HEPES pH 7.5, 100 mM NaCl, 1 mM DTT, 0.1 mg/mL hen egg white lysozyme, supplemented with the cOmplete EDTA-free protease inhibitor cocktail (Roche, Basel, Switzerland) (25 mL of buffer per 1 L culture). Cells were lysed via a single freeze-thaw cycle and sonication and the soluble fraction collected via centrifugation at 30,000g and 4°C for 45 min. The soluble fraction was filtered through 0.45-μm syringe filters and applied to an NiN TA resin equilibrated in 40 mM HEPES, 400 mM NaCl, 20 mM imidazole, 1 mM DTT, pH 7.5 and the column washed in 15 column volumes of the same buffer. Bound protein was eluted with the same buffer containing 300 mM imidazole and directly applied to an amylose resin equilibrated in 20 mM HEPES, 400 mM NaCl, 1 mM DTT, pH 7.5, the column washed with 15 column volumes of the same buffer and eluted in 20 mM HEPES, 400 mM NaCl, 1 mM DTT, 10 mM maltose, pH 7.5. The protein was then concentrated to 2 mL in a 30 kDa MWCO spin concentrator and further purified on a HiLoad Superdex 200 pg 16/600 column equilibrated in 20 mM HEPES pH 7.4, 150 mM NaCl. Fractions assessed to contain pure fusion protein, via SDS-PAGE, were pooled and concentrated to 10-15 mg/mL. Glycerol to 10% (v/v) and 1 mM DTT were added to the concentrate before aliquoting, freezing in liquid N_2_ and storage at -80°C.

1. Eriksen MS, Nikolaienko O, Hallin EI, Grødem S, Bustad HJ, Flydal MI, et al. Arc self-association and formation of virus-like capsids are mediated by an N-terminal helical coil motif. FEBS J. 2020;288:2930-55. PubMed PMID: 33175445.

2. Busso D, Delagoutte-Busso B, Moras D. Construction of a set Gateway-based destination vectors for high-throughput cloning and expression screening in Escherichia coli. Anal Biochem. 2005;343:313-21. doi: 10.1016/j.ab.2005.05.015. PubMed PMID: 15993367.

3. Hallin EI, Eriksen MS, Baryshnikov S, Nikolaienko O, Grødem S, Hosokawa T, et al. Structure of monomeric full-length ARC sheds light on molecular flexibility, protein interactions, and functional modalities. J Neurochem. 2018;147:323-43. doi: 10.1111/jnc.14556. PubMed PMID: 30028513.
