# Supplementary material for: High-affinity anti-Arc nanobodies provide tools for structural and functional studies
Source: PLoS One. 2022 Jun 7;17(6):e0269281. doi: 10.1371/journal.pone.0269281 (PMC9173642; doi:10.1371/journal.pone.0269281)
Supplement: S2 Protocol — (DOCX) [file pone.0269281.s019.docx]

**S2 Protocol**

***Small angle X-ray scattering data collection***

SAXS data from MBP-2rNT, and FLrArc-7A in the presence and absence of nanobodies, were collected on the SWING beamline of Soleil synchrotron, Saint Aubin, France in HPLC mode [1] using a wavelength (λ) of 1.03 Å and a sample-to-detector (EIGER 4X M) distance of 1.8 m, resulting in a momentum transfer (q) range of 0.005-0.5 Å^-1^ (q = 4π sin(θλ^-1^); 2θ is the scattering angle). In the SEC-SAXS setup, the samples run through a SEC column and into the capillary where X-ray scattering is measured. This method allows the collection of hundreds of scattering curves from the SEC peak that corresponds to the pure FLrArc-7A+Nb complexes and thus allows the removal of aggregates and unbound Nb. Nbs were mixed with FLrArc-7A in 1.4-fold molar excess, for a final complex concentration of 7 mg/mL prior to injection, and 50 µl of sample were injected onto a Bio SEC-3 300 column (Agilent Technologies) pre-equilibrated with the running buffer (20 mM HEPES, 150 mM NaCl, 0.5 mM TCEP, pH 7.5). The flow rate was 0.3 ml/min and data were collected with an exposure time of 990 ms and a dead time of 10 ms. Scattering of MBP-2rNT was measured in the same manner at a concentration of 8.3 mg/mL. Buffer data were collected at the beginning of the chromatogram and sample data were acquired in the peak area. Data reduction, R_g_ evaluation over elution profiles, data averaging and merging were performed using the beamline software Foxtrot (version 3.5.2).

SAXS data from hArc-CTD in the absence and presence of Nbs were collected on the BM29 beamline of European Synchrotron Radiation Facility, Grenoble, France in HPLC mode [2]. The data were recorded at a wavelength (λ) of 0.992 Å and a sample-to-detector (PILATUS 1M) distance of 2.867 m, covering a momentum transfer (q) range of 0.004-0.5 Å^-1^ (q = 4π sin θλ^-1^; 2θ is the scattering angle) [2]. Nbs were mixed with hArc-CTD in 1.3-fold molar excess for a final complex concentration of 10 mg/mL and 50 µl of the mixture injected onto an AdvanceBio SEC 130 column (Agilent Technologies) pre-equilibrated with the running buffer (20 mM HEPES, 150 mM NaCl, 0.5 mM TCEP, pH 7.5). Buffer data were collected at the beginning of the chromatogram and sample data were acquired in the peak area. Normalization and radial averaging were performed using the automated pipeline at the beamline [3].

Frame selection and buffer subtraction were carried out in CHROMIXS [4], primary analysis in PRIMUS [5] and distance distribution function (P(r)) analysis in GNOM [6]. *Ab initio* models were created using DAMMIN [7] and GASBOR [8]. Oligomer models were built from individual subunit models based on SAXS data in CORAL [9]. Theoretical scattering curves of coordinate files were calculated using CRYSOL [10] and scattering-based normal mode analysis of crystal structures carried out in SREFLEX [11].

1. David G, Perez J. Combined sampler robot and high-performance liquid chromatography: a fully automated system for biological small-angle X-ray scattering experiments at the Synchrotron SOLEIL SWING beamline. Journal of Applied Crystallography. 2009;42(5):892-900. doi: doi:10.1107/S0021889809029288.

2. Pernot P, Round A, Barrett R, De Maria Antolinos A, Gobbo A, Gordon E, et al. Upgraded ESRF BM29 beamline for SAXS on macromolecules in solution. Journal of Synchrotron Radiation. 2013;20(4):660-4. doi: doi:10.1107/S0909049513010431.

3. Brennich ME, Kieffer J, Bonamis G, De Maria Antolinos A, Hutin S, Pernot P, et al. Online data analysis at the ESRF bioSAXS beamline, BM29. Journal of Applied Crystallography. 2016;49(1):203-12. doi: doi:10.1107/S1600576715024462.

4. Panjkovich A, Svergun DI. CHROMIXS: Automatic and interactive analysis of chromatography-coupled small-angle X-ray scattering data. Bioinformatics. 2018;34:1944-6. doi: 10.1093/bioinformatics/btx846. PubMed PMID: 29300836.

5. Konarev PV, Volkov VV, Sokolova AV, Koch MHJ, Svergun DI. PRIMUS: A Windows PC-based system for small-angle scattering data analysis. J Appl Crystallogr. 2003;36:1277-82. doi: 10.1107/S0021889803012779.

6. Svergun DI. Determination of the regularization parameter in indirect-transform methods using perceptual criteria. J Appl Crystallogr. 1992;25:495-503. doi: 10.1107/S0021889892001663.

7. Svergun DI. Restoring low resolution structure of biological macromolecules from solution scattering using simulated annealing. Biophys J. 1999;76:2879-86. doi: 10.1016/S0006-3495(99)77443-6. PubMed PMID: 10354416.

8. Svergun DI, Petoukhov MV, Koch MHJ. Determination of domain structure of proteins from x-ray solution scattering. Biophys J. 2001;80:2946-53. doi: 10.1016/S0006-3495(01)76260-1. PubMed PMID: 11371467.

9. Petoukhov MV, Franke D, Shkumatov AV, Tria G, Kikhney AG, Gajda M, et al. New developments in the ATSAS program package for small-angle scattering data analysis. J Appl Crystallogr. 2012;45:342-50. doi: 10.1107/S0021889812007662. PubMed PMID: 25484842.

10. Svergun DI, Barberato C, Koch MH. CRYSOL - A program to evaluate X-ray solution scattering of biological macromolecules from atomic coordinates. J Appl Crystallogr. 1995;28:768-73. doi: 10.1107/S0021889895007047.

11. Panjkovich A, Svergun DI. Deciphering conformational transitions of proteins by small angle X-ray scattering and normal mode analysis. Phys Chem Chem Phys. 2016;18:5707-19. doi: 10.1039/c5cp04540a. PubMed PMID: 26611321.
