# Supplementary material for: High-affinity anti-Arc nanobodies provide tools for structural and functional studies
Source: PLoS One. 2022 Jun 7;17(6):e0269281. doi: 10.1371/journal.pone.0269281 (PMC9173642; doi:10.1371/journal.pone.0269281)
Supplement: S3 Protocol — (DOCX) [file pone.0269281.s020.docx]

***S3 Protocol***

***Crystallisation and structure solution of individual proteins and complexes***

NbArc-E5. Anti-Arc Nb E5 was crystallised at 9.6 mg/mL under 1.26 M (NH_4_)_2_SO_4_, 0.1 M LiSO_4_, 0.1 M Tris-HCl pH 7.5 in a 2:1 300-nL drop over a 70 μL reservoir. Rod-shaped crystals in the tetragonal P4_3_2_1_2 spacegroup, with approximate dimensions of 300x40x40 μm^3^, grew after around 30 days at 20°C. Crystals were cryoprotected in the reservoir solution supplemented with 25% (v/v) glycerol. Diffraction data were collected at the I03 beamline at the Diamond light source (Oxford, UK) on an Eiger2 XE 16M detector at an X-ray wavelength of 0.976 Å (12.7 keV) with a 20x20 μm^2^ focused beam at 90.21% transmission. 1700 images were collected with an oscillation of 0.1° (for a total of 170°) with 50 ms exposures. Phases were solved using MR with 5HVG [[1](#_ENREF_1)] as the reference model, for a translation function Z-score (TFZ) of 26.1.

rArc-CTD in complex with NbArc-H11 and -C11. A complex of the FLrArc-7A with Nbs H11 and C11 was prepared as described above, concentrated to 16 mg/mL and 150 μM Stg added to the concentrate prior to crystallisation. Needle-shaped crystals (200-400 μm in length) in the orthorombic P2_1_2_1_2_1_ spacegroup grew in 10% PEG10000, 10% PEG8000 after 5 months at 20 °C, in 1:1, 300-nL drop over a 70 μL reservoir. The crystals were cryoprotected in the reservoir supplemented with 25% PEG400. Diffraction data were collected at the EMBL/DESY P14 beamline of the PETRAIII synchrotron (Hamburg, Germany) on an EIGER 16M detector with an X-ray wavelength of 0.976 Å (12.7 keV). 2000 images were collected with an oscillation of 0.1° (for a total of 200°) and 10 ms exposure with an 150x61 μm^2^ flat beam at 100% transmission. As the crystals turned out to only contain the C-terminal domain of rArc in complex with the Nbs, phases were solved by initially obtaining a partial MR solution by using a crystal structure of the N-lobe of rArc-CTD in complex with Stg (4X3H; [[2](#_ENREF_2)]), the C-lobe of rArc-CTD (4X3X; [[2](#_ENREF_2)]) and a C11 homolog (6H16; [[3](#_ENREF_3)]) as the reference models, giving a TFZ-score of 12.4. The final phases were obtained *via* MR, using a H11 homolog (4FHB, [[4](#_ENREF_4)]) as the reference model and the partial solution from the earlier run, giving a TFZ-score of 17.4. The MR solution was subjected to automatic model building and density modification in AutoBuild within Phenix [[5](#_ENREF_5)], followed by manual model building and refinement.

hArc-CTD in complex with NbArc-H11 and -C11. A complex of hArc-CTD and Nbs H11 and C11 was prepared as described above. Crystals with approximate dimensions of 150x50x50 μm^3^ in the P2_1_2_1_2_1_ space group grew from 37 mg/mL of the complex after around three weeks under 20% PEG3350, 0.2 M Na malonate, 0.2 M KBr at 8 °C in a 1:1, 300-nL drop seated over a 70 μL reservoir. Crystals from the drop were cryoprotected in the reservoir solution supplemented with 25% PEG400. Diffraction data were collected at the P11 beamline [[6](#_ENREF_6)] of the PETRA-III synchrotron (Hamburg, Germany) on a Pilatus 6M detector at an X-ray wavelength of 1.033 Å (12.0 keV). 2000 frames were collected with 0.1° oscillation (for a total of 200°) with a 20x20 μm2 focused beam at 2% transmission and an exposure time of 40 ms. Phases were solved via MR, using the hArc N-lobe in complex with Stg, C-lobe of hArc (6TNO and 6TN7, respectively;[[7](#_ENREF_7)]) and the H11 and C11 Nbs from the rArc structure as the reference models, giving a TFZ-score of 17.8. The MR solution was run through AutoBuild [[5](#_ENREF_5)], before manual model building and refinement. Efforts to solve the phases using the rArc-CTD-Nb crystal structure were unsuccessful.

hArc-CTD in complex with NbArc-H11 and -C11, collapsed crystal form. The ternary complex of hArc-CTD with Nbs H11 and C11 was prepared as described above and concentrated to 10.0 mg/mL. Microseeds of crystals of the same complex were prepared from crystals grown under 17% PEG3350, in a 1:1 300 nL drop, as described above. Crystals were grown using the hanging drop vapour diffusion method in drops consisting of 1 μL protein, 0.25 μL 1,024-fold diluted seed stock and 0.75 μL reservoir (12% PEG3350), hanging over 500 μL of the same reservoir solution. Needle shaped crystals in the orthorhombic P2_1_2_1_2 spacegroup grew after a 3-month incubation at 20°C. Crystals were cryoprotected in the reservoir solution supplemented with 25% PEG400. Diffraction data were collected on the P11 beamline at DESY [[6](#_ENREF_6)], with an X-ray wavelength of 1.033 Å on a Dectris Eiger 16M detector. 3600 frames were collected with an oscillation of 0.1°/frame (360° total) using a 50x50 μm^2^ focused beam with an exposure time of 10 ms and 35% transmission. Due to an underestimation of the resolution limit upon collection, the data were collected with the detector edge at 2.4 Å. However, upon processing the data it became clear that the data extended much further and utilising the corners of the detector the data could be processed to 1.94 Å, but to lower than ideal completeness. Phases were solved using MR. Inititally, only the structure of H11 from the earlier hArc complex was used as the reference model, giving a TFZ score of 18.9, followed by the addition of the crystal structure of the hArc N-lobe in complex with Stg (6TNP; [[7](#_ENREF_7)]), with Stg deleted from the model, for a TFZ score of 28.6. Finally, the C11 Nb structure from the earlier complex was used as the reference model, using a partial solution from the earlier run, to give a TFZ score of 31.9. Phases could not be solved using the C-lobe of hArc as a reference model, so what remained of the C-lobe was built into the model manually.

For matrix micro-seeding experiments, crystal seeds were produced by crushing protein crystals with a Pasteur pipette, end of which had been shaped into a ball using a flame. 4 μL of the reservoir solution were pipetted onto the crystal fragments and moved into a Seed Bead Eppendorf tube (Hampton research). This was repeated until the accumulated volume reached 28 µL, to create the undiluted seed stock. The seed stock was vortexed in 10 s intervals for 1-2 min and serially diluted four-fold to a 4,096x dilution. Crystallisation *via* seeding was carried out in 48-well hanging drop plates, with 2 μL drops, consisting of 1 μL protein, 0.75 μL reservoir and 0.25 μL of the appropriately diluted seed stock, over 200 μL reservoirs.

1. Zhou X, Weeks SD, Ameloot P, Callewaert N, Strelkov SV, Declerck PJ. Elucidation of the molecular mechanisms of two nanobodies that inhibit thrombin-activatable fibrinolysis inhibitor activation and activated thrombin-activatable fibrinolysis inhibitor activity. J Thromb Haemost. 2016;14:1629-38. doi: 10.1111/jth.13381. PubMed PMID: 27279497.

2. Zhang W, Wu J, Ward MD, Yang S, Chuang YA, Xiao M, et al. Structural basis of arc binding to synaptic proteins: Implications for cognitive disease. Neuron. 2015;86:490-500. doi: 10.1016/j.neuron.2015.03.030. PubMed PMID: 25864631.

3. Fenderico N, van Scherpenzeel RC, Goldflam M, Proverbio D, Jordens I, Kralj T, et al. Anti-LRP5/6 VHHs promote differentiation of Wnt-hypersensitive intestinal stem cells. Nat Commun. 2019;10:1-13. doi: 10.1038/s41467-018-08172-z. PubMed PMID: 30664649.

4. Oyen D, Wechselberger R, Srinivasan V, Steyaert J, Barlow JN. Mechanistic analysis of allosteric and non-allosteric effects arising from nanobody binding to two epitopes of the dihydrofolate reductase of Escherichia coli. BBA-Proteins Proteom. 2013;1834:2147-57. doi: 10.1016/j.bbapap.2013.07.010. PubMed PMID: 23911607.

5. Liebschner D, Afonine PV, Baker ML, Bunkoczi G, Chen VB, Croll TI, et al. Macromolecular structure determination using X-rays, neutrons and electrons: Recent developments in Phenix. Acta Crystallogr D. 2019;75:861-77. doi: 10.1107/S2059798319011471. PubMed PMID: 31588918.

6. Burkhardt A, Pakendorf T, Reime B, Meyer J, Fischer P, Stübe N, et al. Status of the crystallography beamlines at PETRA III. Eur Phy J Plus. 2016;131:1-9. doi: 10.1140/epjp/i2016-16056-0.

7. Hallin EI, Bramham CR, Kursula P. Structural properties and peptide ligand binding of the capsid homology domains of human Arc. Biochem Biophys Rep. 2021;26:100975. doi: 10.1016/j.bbrep.2021.100975.
